# Immuno-profiling and cellular spatial analysis using five immune oncology multiplex immunofluorescence panels for paraffin tumor tissue

**DOI:** 10.1038/s41598-021-88156-0

**Published:** 2021-04-19

**Authors:** Edwin Roger Parra, Maria C. Ferrufino-Schmidt, Auriole Tamegnon, Jiexin Zhang, Luisa Solis, Mei Jiang, Heladio Ibarguen, Cara Haymaker, J. Jack Lee, Chantale Bernatchez, Ignacio Ivan Wistuba

**Affiliations:** 1 Department of Translational Molecular Pathology, Unit 951, The University of Texas MD Anderson Cancer Center, 2130 Holcombe Blvd., Houston, TX 77030 USA; 2 Department of Bioinformatics and Computational Biology, The University of Texas MD Anderson Cancer Center, Houston, TX USA; 3 Department of Biostatistics, The University of Texas MD Anderson Cancer Center, Houston, TX USA; 4 Department of Melanoma Medical Oncology, The University of Texas MD Anderson Cancer Center, Houston, TX USA

**Keywords:** Cancer, Immunology, Biomarkers, Medical research

## Abstract

Multiplex immunofluorescence (mIF) has arisen as an important tool for immuno-profiling tumor tissues. We updated our manual protocol into an automated protocol that allows the use of up to seven markers in five mIF panels to apply to formalin-fixed paraffin-embedded tumor tissues. Using a tyramide signal amplification system, we optimized five mIF panels that included cytokeratin to characterize malignant cells (MCs), immune checkpoint markers (i.e., PD-L1, B7-H3, B7-H4, IDO-1, VISTA, LAG3, ICOS, TIM3, and OX40), tumor-infiltrating lymphocytic markers (i.e., CD3, CD8, CD45RO, granzyme B, PD-1, and FOXP3), and markers to characterize myeloid-derived suppressor cells (i.e., CD68, CD66b, CD14, CD33, Arg-1, and CD11b). To determine analytical reproducibility and the impact of those panels for immuno-profiling tumor tissues, we performed an exploratory analysis in a set of non–small cell lung cancer (NSCLC) samples. The slides were scanned, and the different cell phenotypes were quantified by simultaneous co-localizations with the markers using image analysis software. Comparison between the time points of staining showed high analytical reproducibility. The analysis of NSCLC cases showed an immunosuppressive microenvironment with PD-L1/PD-1 expression as a predominant axis. Interestingly, high density of MCs expressing B7-H4 was correlated with recurrence. Unexpectedly, MCs expressing OX40 were also detected, and those cells were a closer distance to CD3+T-cells than were MCs expressing other immune checkpoints. Two different cellular patterns of spatial distribution were determined according the CD3 distribution, and the predominant pattern was related with active immunosuppressive interaction with MCs. Our study shows that these five mIF panels can identify multiple targets in a single cell with high reproducibility. The study of different cell populations and their spatial relationship can open new ideas for therapeutic approaches.

## Introduction

Immunotherapy for cancer is developing constantly, with promising results. However, aggressive tumors are able to escape immune surveillance by creating an immunosuppressive microenvironment in which the tumor thrives^1,2^. Since the description of cytotoxic T-lymphocyte-associated protein 4 (CTLA-4) and programmed cell death protein 1 (PD-1) and their roles in tumor immune evasion^3,4^, understanding the interaction between immune cells and cancer cells has become increasingly important as a starting point for the development of new immunotherapies^5–7^. Acquiring this knowledge demands the development of new technologies that allow researchers to study newly identified molecules and their interactions^8^.

Multiplex immunofluorescence (mIF) is a reliable high-throughput method that allows cell-by-cell identification of multiple markers on tumor cells and tumor-associated immune cells^9^. Because mIF allows the use of up to seven markers in one slide^10^, we can directly observe various biomarkers expressed by a single cell and analyze their spatial relationships in various cell populations, something that cannot be achieved by traditional chromogen-based immunohistochemistry (IHC)^11–13^. Thus, with a combination of carefully selected antibodies, many cell subsets can be identified. In our first study, we described the optimization of two mIF panels, six markers each, using tyramide signal amplification (TSA) in a manual protocol as a standard reference to validate the multiplex staining^14^. The updated method for these two panels described here has been developed by using a commercially available Opal fluorophores in an automated stainer that drastically decreased our first manual protocol staining time from 4 to 5 days to 14 to 17 h and improved the consistency of the staining. The resulting improvement in the immunological comprehension of those panels led us to change some markers in the panels. In addition, we developed three new mIF panels, seven markers each, to characterize key cell populations as immune checkpoint and myeloid suppressor phenotypes in the tumor microenvironment for formalin-fixed, paraffin-embedded tumor tissues. We show the optimization and reproducibility of these automated immuno-oncologic panels and their application to study the tumor microenvironment and spatial distribution of cell phenotypes in a small cohort of non-small cell lung cancer (NSCLC) samples.

## Materials and methods

### Tissue specimens

As described previously^14,15^, sequential 4-µm-thick sections from a tissue microarray that included formalin-fixed, paraffin-embedded lung cancer control tissues as well as reactive human tonsil tissues were prepared for optimization of conventional IHC, optimization of single immunofluorescence (single IF), and both optimization and reproducibility of mIF for all antibodies included in this study (Supplementary Table 1). Additionally, sequential 4-µm-thick sections from 10 samples of NSCLC, including five adenocarcinomas (ADCs) and five squamous cell carcinomas (SCCs), were prepared for mIF staining and analysis. All available clinicopathologic information from the NSCLC cohort was retrieved from the electronic clinical records of those patients (Supplementary Table 2) and included age, sex, smoking history, pathologic TNM stage according to the 8th edition of the American Joint Committee on Cancer staging system^16^, adjuvant treatment, and follow-up information for recurrence and vital status. These characteristics were later assessed for possible correlations with the mIF data.

**Table 1:** The frequently observed phenotypes in the five multiplex immunofluorescence panels in the non-small cell lung cancer cohort (N = 10).

Panel	Marker co-expression	Phenotype	Median density, cells/mm^2^
1	CK+	All malignant cells	3192.23
	CK+PD-L1+	All malignant cells expressing PD-L1	123.15
	CD3+	All T lymphocytes	520.78
	CD3+CD8+	Cytotoxic T-cells	376.98
	CD3+PD-1+	Antigen experienced T-cells	26.01
	CD3+CD8+PD-1+	Cytotoxic T-cells antigen experienced	17.94
	CD68+	All tumor associated macrophages (TAMs)	120.48
	CD68+PD-L1+	TAM expressing PD-L1	82.88
2	CK+	All malignant cells	2777.47
	CD3+	All T lymphocytes	591.10
	CD3+CD8+	Cytotoxic T-cells	324.76
	CD3+CD8+GBZ+	Activated cytotoxic T-cells	21.20
	CD3+CD45RO+	Memory T-cells	958.21
	CD3+CD8+CD45RO+	Effector/memory T-cells	114.81
	CD3+FOXP3+CD8-	Regulatory T-cells	134.53
3	CK+	All malignant cells	2607.35
	CK+PD-L1+	Malignant cells expressing PD-L1	166.33
	CK+B7-H3+	Malignant cells expressing B7-H3	0.23
	CK+B7-H4+	Malignant cells expressing B7-H4	4.71
	CK+IDO-1+	Malignant cells expressing IDO-1	77.42
	CD3+	All T lymphocytes	477.22
	CD3+PD-L1+	T-cells expressing PD-L1	58.58
	CD3+B7-H3+	T-cells expressing B7-H3	426.26
	CD3+B7-H4+	T-cells expressing B7-H4	0.00
	CD3+IDO-1+	T-cells expressing IDO-1	9.42
	CD68+	All TAMs	115.99
	CD68+PD-L1+	TAM expressing PD-L1	66.71
	CD68+B7-H3+	TAM expressing B7-H3	0.01
	CD68+B7-H4+	TAM expressing B7-H4	0.00
	CD68+IDO-1+	TAM expressing IDO-1	16.49
4	CK+	All malignant cells	2985.61
	CD3+	All T lymphocytes	432.36
	CD3+VISTA+	T-cells expressing VISTA	10.60
	CD3+ICOS+	T-cells expressing ICOS	35.33
	CD3+LAG3+	T-cells expressing LAG3	6.77
	CD3+OX-40+	T-cells expressing OX-40	11.48
	CD3+TIM3+	T-cells expressing TIM3	1.18
5	CK+	All malignant cells	2949.70
	CD68+	All TAMs	92.14
	CD68+Arg-1+	TAM type II	0.12
	CD68+CD11b+	Dendritic macrophages	82.72
	CD11b+CD66+	Polymorphonuclear leukocyte (PMN)	8.83
	CD11b+Arg-1+CD14+CD33+	Monocytic myeloid-derived suppressor cells (MDSC-M)	0.02
	CD11b+CD66b+CD33+	Granulocytic myeloid-derived suppressor cells (MDSC-PMN)	1.23

This study was approved by the MD Anderson Institutional Review Board. Informed consent to participate was obtained from all participants included in this study, and all methods were performed in accordance with the relevant guidelines and regulations and are available for review at any time.

### IHC optimization

IHC optimization was performed using an automated staining system (BOND-MAX; Leica Biosystems, Vista, CA) with previously optimized and validated antibodies^15,17,18^ against cytokeratin (CK) to characterize malignant cells (MCs), immune checkpoint markers (i.e., PD-L1, B7-H3, B7-H4, IDO-1, VISTA, LAG3, ICOS, TIM3, and OX40), tumor-infiltrating lymphocyte markers (i.e., CD3, CD8, CD45RO, granzyme B, PD-1, and FOXP3), and markers to characterize myeloid-derived suppressor cells (i.e., CD68, CD66b, CD14, CD33, Arg-1, and CD11b) (Supplementary Table 1). Expression of all cell markers was detected using a Novocastra BOND Polymer Refine Detection Kit (Leica Biosystems, #DS9800), with a diaminobenzidine reaction to detect antibody labeling and hematoxylin counterstaining. To obtain uniform staining, several tests were performed using different antibody dilutions and antigen retrieval conditions until optimal conditions were obtained for the primary and secondary antibody in the positive tonsil controls. Antibody clones, vendors, and final IHC dilutions are shown in Supplementary Table 1.

### Single IF antibody optimization

To update and create the mIF panels, we grouped the antibodies optimized and tested by IHC into five immuno-oncology panels containing 4′,6-diamidino-2-phenylindole (DAPI) plus six or seven antibodies each, including updated *panel 1*: CK, CD3, CD8, PD-1, PD-L1, CD68, and 4′,6-diamidino-2-phenylindole (DAPI); updated *panel 2*: CK, CD3, CD8, CD45RO, granzyme B, FOXP3, and DAPI; new *panel 3*: CK, CD3, PD-L1, B7-H3, B7-H4, IDO-1, CD68, and DAPI; new *panel 4*: CK, CD3, ICOS, LAG3, OX40, TIM3, VISTA, and DAPI; and new *panel 5*: CK, Arg-1, CD11b, CD14, CD33, CD66b, CD68, and DAPI (Supplementary Table 3). For single IF optimization, all the antibodies from each panel were assessed using the same positive controls as in the IHC optimization, stained using an autostainer (Leica BOND RX, Leica Biosystems), and linked with a fluorophore from the Opal 7 color IHC kit (#NEL797001KT; Akoya Biosciences, Waltham, MA), including DAPI and Opal Polaris 520, 540, 570, 620, 650, and 690. For the new panels with seven antibodies, the TSA fluorophore Opal Polaris 480 (#FP1500001KT, Akoya Biosciences) was added to the kit. For single IF protocols, after baking and dewaxing (BOND Dewax Solution, Leica Biosystems), the slides were heated at 95 °C for 20 min using Bond Antigen Retrieval Tris–ethylenediaminetetraacetic acid buffer or citrate buffer according to the conditions previously determined by IHC (Supplementary Table 3) to open antibody epitopes. Next, the slides were incubated between 30 and 60 min at room temperature, depending on the antibody, at dilutions similar to those used for IHC staining (Supplementary Table 1). The slides were washed three times with 1 × 2-methyl-2H-isothiazol-3-one (#AR9590, BOND Wash Solution, Leica Biosystems) and then incubated for 10 min at room temperature with polymer horseradish peroxidase conjugated to anti-mouse/rabbit secondary antibody (Akoya Biosciences). After five successive washes with BOND Wash Solution, the slides were incubated for 10 min with an Opal fluorophore tyramide (Opal Polaris 480, 520, 540, 570, 620, 650, or 690) and prepared according to the manufacturer’s instructions at dilutions of 1:50 to 1:150 to detect the different antibodies. After four additional washes with BOND Wash Solution, the slides were counterstained with DAPI for 5 min. The slides were removed from the autostainer and manually mounted with ProLong Diamond Antifade Mountant (Thermo Fisher Scientific, Waltham, MA). For each run of staining, three types of autofluorescence (negative control) slides were run in parallel: 1) primary and secondary antibodies; 2) Opal fluorophore tyramides and secondary antibodies; and 3) only secondary antibodies. Negative controls allowed the extraction of endogenous and exogenous autofluorescence from the tissues. Several tests were done combining antibodies and Opal fluorophores until a specific signal from each antibody was recognized; Supplementary Table 3 shows the final combination of antibodies and Opal fluorophores.

### Expected cell phenotype characterization using the five mIF panels

The two updated panels and the three new panels we created showed a high ability to identify individual markers and co-localization of various biomarkers in the same cell, thus characterizing specific cell phenotypes in the tumor microenvironment. According to the co-expression of markers shown in Table 1, the panels were designed to label specific cell populations as follows: *panel 1:* for the axis of PD-L1/PD-1 and T-cells, *panel 2*: for activation and regulation of T-cells, *panel 3*: for immune checkpoint markers expressed by MCs, *panel 4*: for costimulatory and inhibitory immune checkpoint markers mostly expressed by T-cells, and *panel 5*: for myeloid suppressor cell phenotypes.

### Spectral library

A spectral library was created for multispectral image analysis visualization and fluorophore extraction. Control tissues were stained using a CD20 antibody (B-cell marker, clone L26, dilution 1:100, Dako) as an abundant expression marker in the tonsil linked to one of the eight Opal fluorophore tyramides, following conditions similar to those used for IHC but without DAPI to obtain abundant signal with each of the fluorophores (Supplementary Fig. 1)^19^.

**Figure 1:**
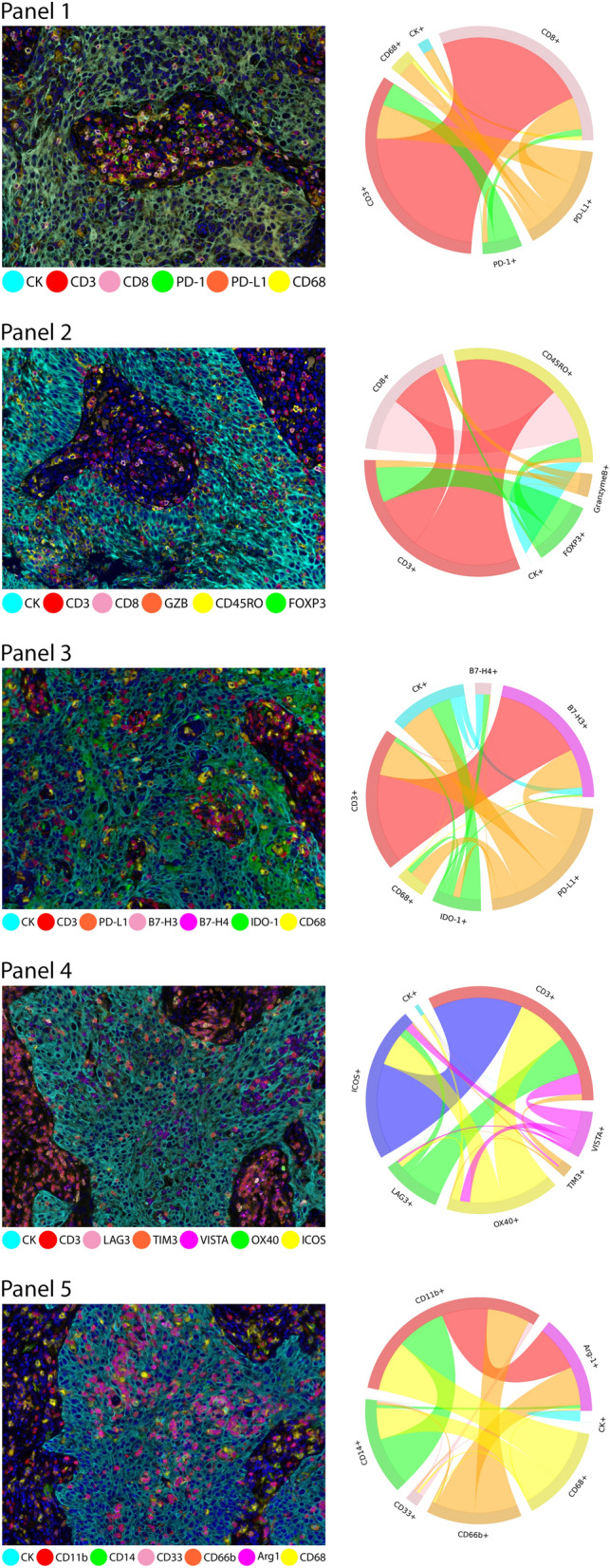
Microphotographs of representative examples of multiplex immunofluorescence (mIF) and cord plots of cells co-expression of makers in the different immuno-oncology panels in the NSCLC cohort. For each panel, the composite mIF images (left) and the diversity of cell phenotypes by the markers co-expressed (right) are shown. mIF 20 × magnification. The images were generated using Vectra-Polaris 3.0.3 scanner system and InForm 2.4.8 image analysis software (Akoya Biosciences) and R studio software version 3.6.1.

### mIF optimization

Once each target was optimized using a single IF protocol, the protocols were combined to obtain the mIF protocol for the different panels. The five panels were used to stain human tonsil specimens as positive controls. Staining for the different markers was performed consecutively using the same steps as those used in single IF, and detection for each marker was completed before application of the next antibody. Using automated protocols, we set up the sequence of antibodies in each panel and tested each sequence several times until we obtained the same staining pattern as was shown in the single IF. Dynamic ranges^19^ from the different antibodies linked with their particular fluorophore were set up to obtain similar ranges of expression, with 50–150 ns of exposure time, for each antibody, determined by the Vectra-Polaris 3.0.3 scanner system (Akoya Biosciences). The dynamic range of each antibody was carefully adjusted to avoid a cross-talking reaction^20^ between fluorophores or an umbrella effect in which the expression of one antibody is blocking the expression of another that is expressed in the same cell compartment^19^. For each run of multiplex staining, three types of autofluorescence (negative control) slides were run in parallel.

### NSCLC tissue scanning, reproducibility and analysis

To analyze the reproducibility of the different mIF panels, the 10 formalin-fixed, paraffin-embedded NSCLC tumor samples were stained with the panels. Consecutive tissue sections were stained for each panel at two time points with a 1-week interval (week 1 and week 2).

To calibrate the spectral image scanner protocol, the stained slides were scanned using the Vectra-Polaris 3.0.3, a multispectral imaging system (Akoya Biosciences), at a low magnification of 10 × (1.0 μm/pixel) through the full emission spectrum, from 440 to 720 nm, to extract fluorescence intensity information from the images using the tonsil positive controls running in parallel with the samples. The NSCLC samples were then scanned at a high magnification using Vectra-Polaris 3.0.3 (931 × 698 µm size at resolution 20 × , 0.5 µm/pixel), and two pathologists used the Phenochart 1.0.9 image viewer software to select five regions of interest (ROIs) to capture various elements of tissue heterogeneity. To assess analytical reproducibility, we captured each ROI from week 1 and 2 in the same location on the consecutive slide for each panel, thus quantifying the cell phenotypes at the same locations in the specimen between the two time points (Supplementary Fig. 2). The same spectral signature for each fluorophore was obtained using a “spectral unmixing library” in the image analysis software (InForm 2.8.2, Akoya Biosciences), and a histologic analysis was performed for the entire ROI. The various markers were characterized and quantified using the same algorithm for each panel, through cell segmentation and cell phenotyping, from the InForm image analysis software tool. Finally, to obtain the co-expression of markers, cell phenotypes, we merged the individual markers analyzed from each panel using the x and y coordinates of each cell with the phenoptr script from R Studio (Akoya Biosciences). The final report of cell phenotype density was normalized per mm^2^ for each ROI from the various samples and panels, and this report was used for the analysis of the NSCLC samples.

**Figure 2:**
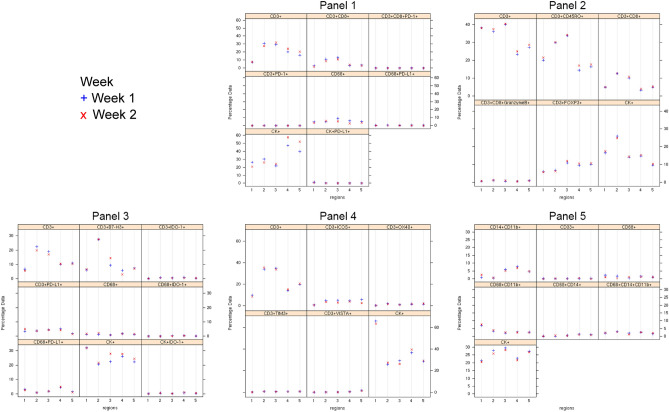
Trellis plots of percentage data from a representative case (ADC-1) by region and week. The blue dots represent week 1 and the red dots represent week 2, visualizing the consistency between the 2 weeks for each cell phenotype obtained by dividing the cell phenotype studded by the total number of cells. The Supplementary Figs. 9 to 13 show the rest of the samples analyzed. The images were generated using R studio software version 3.6.0.

### NSCLC functional spatial distribution analysis

To explore the potential of the data, we analyzed the spatial distribution of the different cell phenotypes observed in the five mIF immune-oncology panels preformed on the NSCLC cohort. The distance between MCs and each cell phenotype included in the panels was calculated using a matrix created with the X and Y coordinates for each cell, which were provided by the image analysis and using the R studio software v.3.6.1. The median distance from MCs to the different cell phenotypes was used to divide the different subpopulations according to their geographical distribution as close to the MCs (CK+) when they were located less than or equal to the median radius distance and far when they were located farther than the median radius distance. To evaluate cell interaction and spatial pattern distribution between MCs and the different cell phenotypes, we compared the empirically derived nearest neighbor distance G function for marker point patterns to the theoretical Poisson function (median distances of the specific cells between samples) obtained by assuming the same intensity of the observed pattern in each case.

### Statistical analysis

To assess the reproducibility of results over time and to determine the potential impact of tumor heterogeneity, percentages representing cell phenotypes were calculated by dividing the density of each phenotype by the density of total nucleated cells (DAPI+) on each consecutive ROI from each panel and for each time point. Specific cell phenotypes observed in each panel from the cohort of 10 NSCLC samples were analyzed, and we assessed the correlation within each sample and across all samples between the two time points using Spearman rank correlation coefficients. The *P*-values obtained for each marker on each panel between two time points were adjusted by Bonferroni correction, and adjusted *P*-values < 0.05 were considered significant. To characterize the analytical reproducibility of the studied cell phenotypes, we calculated the coefficient of variation (CV, number of ROIs × number of samples per panel) between the two time points within each ROI of each sample. For each phenotype on each panel, 50 CVs were analyzed. For the analysis of the NSCLC cohort, the chi-square test or Fisher exact test were used to examine differences in categorical variables, and the Wilcoxon rank-sum test and Kruskal–Wallis test were used to explore differences in continuous variables when comparing the predominant cell phenotype densities in each panel. Furthermore, to determinate the spatial interaction between cell phenotypes and to characterize the patterns of cell distribution, we applied the nearest neighbor distance G function and the theoretical Poisson curve. Statistical analyses were carried out using the R software program (versions 3.6.0 and 3.6.1, released April 2019; https://www.r-project.org/).

### Ethics approval and informed consent to participate

This study was approved by the MD Anderson Institutional Review Board. Informed consent to participate was obtained from all participants included in the study, and all methods were performed in accordance with the relevant guidelines and regulations and are available for review at any time.

## Results

### Marker expression on IHC and mIF in positive controls

As shown in microphotographs of chromogenic IHC and mIF (Supplementary Fig. 3), we successfully optimized the various markers to obtain similar staining patterns with both techniques. As expected, PD-L1+ expression was observed predominantly on the cell membranes of epithelial cells from the tonsil crypts. Likewise, the other immune checkpoint markers—including B7-H3, B7-H4 (tested and observed in MCs from the lung cancer tissue microarray positive control), VISTA, LAG3, ICOS, and TIM3—and the immune checkpoint stimulatory markers such as IDO-1 and OX40—were observed in different immune cells with similar distribution patterns between IHC and mIF in tonsil and other control tissues. In control tonsil tissues, CK was expressed by epithelial cells, and among cells surrounding the germinal centers, the T-cell marker CD3 was the most abundant, followed by CD8, granzyme B, CD45RO, and FOXP3 (nuclear expression). PD-1 was observed predominantly distributed in the germinal center of the tonsil tissues and with a similar pattern on IHC and mIF. Markers included in the myeloid panel, such as the macrophage marker CD68, were localized in the germinal centers of the tonsil, as were CD14 and CD11b. The markers CD33 and CD66b were expressed by cells diffusely distributed around and between germinal centers. Arg-1 was seen in cells in the germinal center and next to epithelium, showing a linear distribution in tonsil control tissues on IHC and mIF.

**Figure 3:**
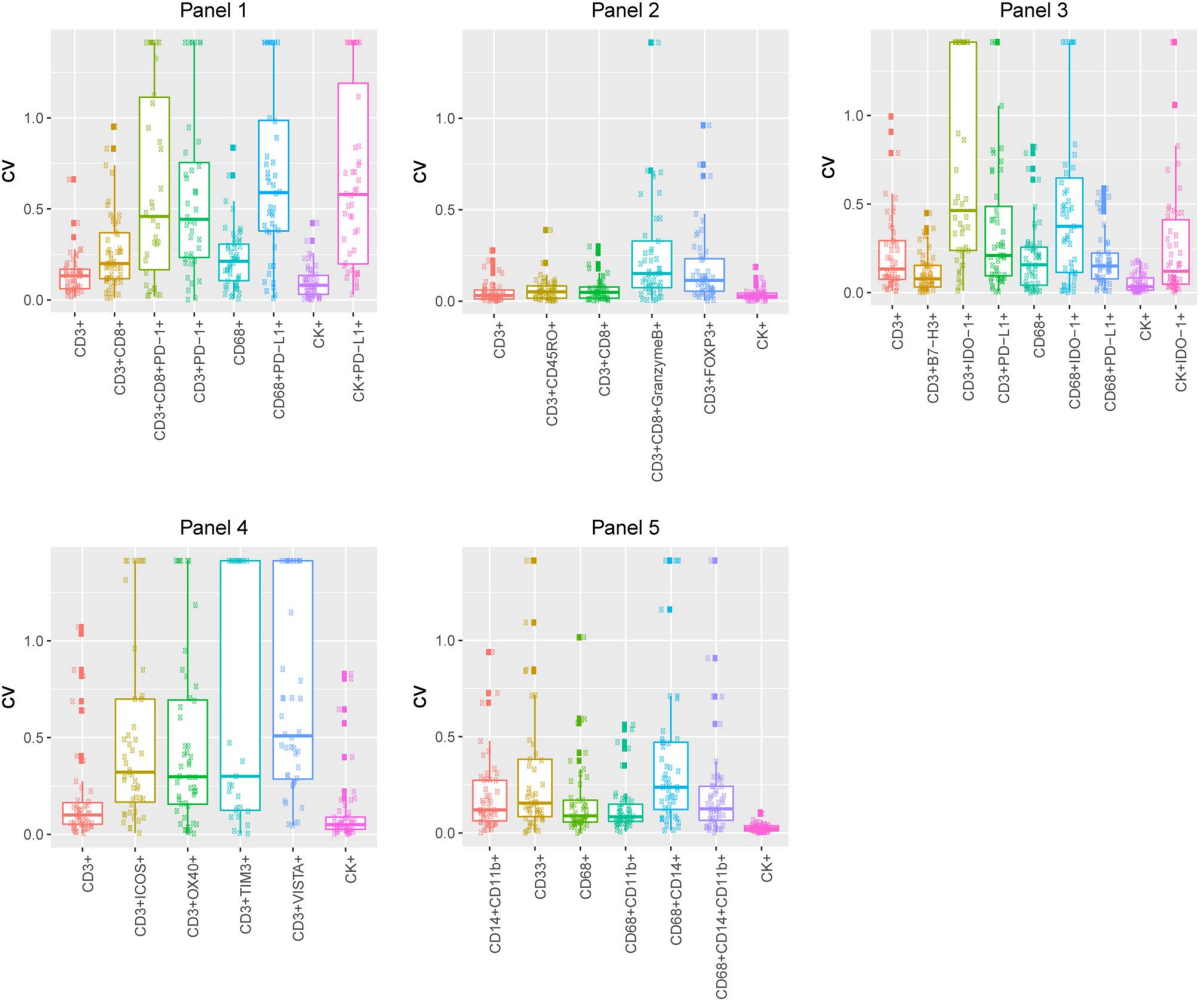
Coefficients of variation (CVs) for each panel. Boxplots show that the median value of the CV, within each region of each sample (at 2 weeks), is less than 0.5 for all the phenotypes studied in all panels. The images were generated using R studio software version 3.6.0.

### Biologic and analytical reproducibility of cell phenotypes

After testing each mIF panel in control tissues and comparing the results with those of IHC for each individual marker, we applied the panels to the 10 NSCLC tissue specimens as shown in the representative images in Fig. 1. We stained two batches, in consecutive weeks, and then compared the most observed biologically relevant cell phenotypes in each panel to assess the reproducibility and variability of the results. The selected phenotypes were: *Panel 1*: CD3+, CD3+CD8+, CD3+CD8+PD-1+, CD3+PD-1+, CD68+, CD68+PD-L1+, CK+, and CK+PD-L1+; *Panel 2*: CD3+, CD3+CD45R0+, CD3+CD8+, CD3+CD8+GranzymeB+, CD3+FOXP3+, and CK+; *Panel 3*: CD3+, CD3+B7-H3+, CD3+IDO-1+, CD3+PD-L1+, CD68+, CD68+IDO-1+, CD68+PD-L1+, CK+, and CK+IDO-1+; *Panel 4*: CD3+, CD3+ICOS+, CD3+OX40+, CD3+TIM3+, CD3+VISTA+, CK+; *Panel 5*: CD14+CD11b+, CD33+, CD68+, CD68+CD11b+, CD68+CD14+, CD68+CD14+CD11b+, and CK+.

In the NSCLC cohort, the phenotypes CD3+CD8+CD45RO+, CK+B7-H3+, CK+B7-H4+, CD3+B7-H4+, CD68+B7-H3+, CD68+B7-H4+, CK+OX40+, CD3+LAG3+, CD68+Arg-1+, CD66b+CD11b+, CD11b+Arg1+CD14+CD33+and CD11b+CD66b+CD33+ were observed in small quantities, and in some samples they were not found; this scarcity hindered the analysis and interpretation of those cell phenotypes, which is why those phenotypes were not included in the reproducibility analysis.

Although the geographic distribution cell phenotypes included in the analysis differed between mIF batches, they still exhibited positive and significant correlations overall between week 1 and week 2 for the different samples and panels (Supplementary Figs. 4 to 8). Indeed, the individual samples demonstrated high reproducibility and consistency of the cell phenotypes included in the panels, among ROIs and between weeks (Fig. 2 and Supplementary Figs. 9 to 13). We found that all studied cell phenotypes had a median CV < 0.5. The CVs were calculated between time points, therefore showing high experimental reproducibility between weeks (Fig. 3). Combining the results from the CVs and the correlation study, we found that in all five panels the analyzed cell phenotypes had a relatively high correlation within samples (median Spearman correlation coefficients > 0.5), except for the phenotypes CD68+ in panel 1, CD3+IDO-1+ in panel 3, and CD3+VISTA+ in panel 4; and between samples (median Spearman correlation coefficients > 0.7), except for CK+ and CK+PD-L1+ in panel 1. Furthermore, when comparing the distribution of the percentages of CK+ (Supplementary Fig. 14) and CD3+T-cells (Supplementary Fig. 15) between the weeks and panels, we observed high correlations between consecutive weeks but not between panels, suggesting that the level of the cut and the selection of ROIs between panels have important impacts in the geographic distribution of the cell phenotypes that characterize tumor heterogeneity in the samples.

**Figure 4:**
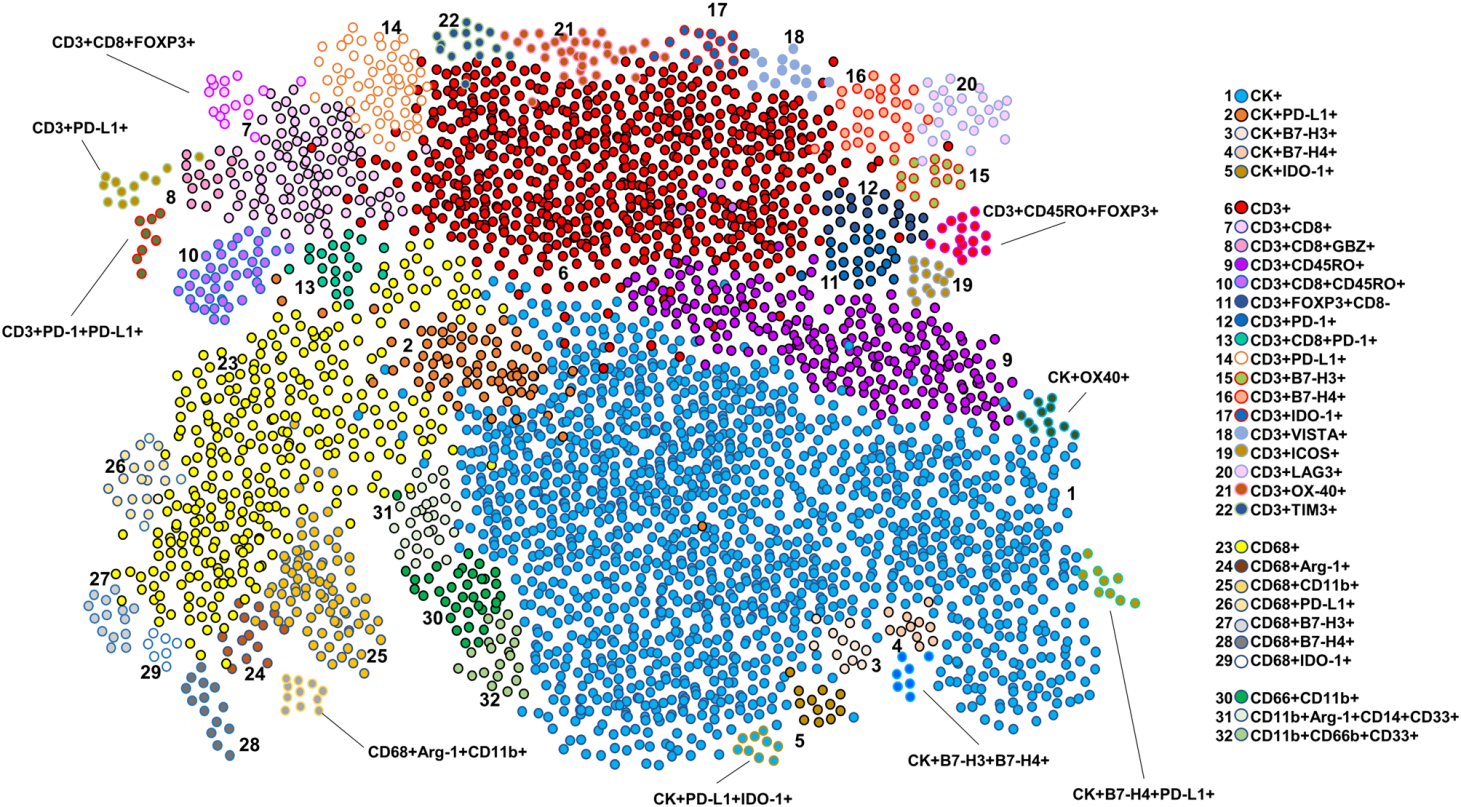
Graphic representation showing the clustering of different cells phenotypes obtained using the five multiplex immunofluorescence panels from a representative case of non-small cell lung cancer. On the right is displaying the list of expected cell phenotypes observed with those panels. Co-expression of different other cell phenotypes are also observed in clustering labeled by the co-expression of markers. The graphic was generated using R studio software version 3.6.1.

### Patients’ clinicopathologic characteristics

Clinicopathologic characteristics are shown in Supplementary Table 2. No patients received neoadjuvant therapy in this cohort, and nine patients are alive at the moment this manuscript is written.

### Characterization of tumor microenvironment in NSCLC

Using the five mIF panels, it was possible to study the tumor microenvironment in our cohort of NSCLC and to identify different tumor-associated immune cell populations, as shown in Table 1 and Fig. 4, using the co-expression of cell-type specific markers. In this cohort, most cases were classified as PD-L1+, nine of 10 were positive (5 ADC and 4 SCC) with a cutoff of greater than 1% of the MCs expressing PD-L1. Additionally, using the same cutoff as PD-L1, we observed seven of 10 cases were positive for IDO-1 (2 ADC and 4 SCC), only 1 SCC was positive for B7-H4, and no cases were positive for B7-H3. However, B7-H4 and B7-H3 expression were observed in low densities in MCs. Although, we saw several MCs that simultaneously expressed immune checkpoint markers, none of those phenotypes were greater than 1%. The predominant CD3+T-cells were memory T-cells (CD3+CD45RO+, median 952.20 cells/mm^2^) and cytotoxic T cells (CD3+CD8, median 350.87 cells/mm^2^). We also observed considerable densities of regulatory T-cells (CD3+FOXP3+, median 134.53 cells/mm^2^) and antigen-experienced T-cells expressing PD-L1 (CD3+PD-L1+ median, 58.58 cells/mm^2^) and B7-H3 (CD3+B7-H3+ median, 426.26 cells/mm^2^), suggesting a potential T-cell–suppressive axis in this group of NSCLC. Looking at macrophages, we observed important numbers of macrophages expressing PD-L1 (CD68+PD-L1+ median, 74.79, cells/mm^2^). Interestingly, patients who had disease recurrence showed higher densities of MCs expressing B7-H4 (median, 4.71 cells/mm^2^) than did patients who did not experience recurrence (median, 2.35 cells/mm^2^, *P* = 0.017). No other significant clinical correlation was observed in this study.

### Identification of other cell populations in NSCLC

Using the five mIF panels, we had the opportunity to observe the co-expression of different cell markers, as shown in Fig. 4, and characterize and explore different cellular subpopulations. Although we observed different combinations of marker co-expression identifying suppressor phenotypes such as CD3+PD-L1+PD-1+, CD3+CD45RO+FOXP3+, and others, as well as a variety of simultaneous combinations of different immune checkpoint markers co-expressed in MCs, such as CK+PD-L1+IDO-1+, CK+B7-H3+B7-H4+, and CK+B7-H4+PD-L1+, those subpopulations were observed in low proportions. Unexpectedly, we identified with panel 4 the expression of OX40 not only in CD3+T-cells but also in MCs (CK+OX40+). Although this phenotype was observed in low densities (CK+OX40+ median, 3.53 cells/mm^2^), most of the tumors, eight of 10 samples (3 ADC and 5 SCC), showed this co-expression, suggesting that CK+OX40+ may be an important marker for future studies.

### Exploratory spatial distribution analysis in NSCLC

**Figure 5:**
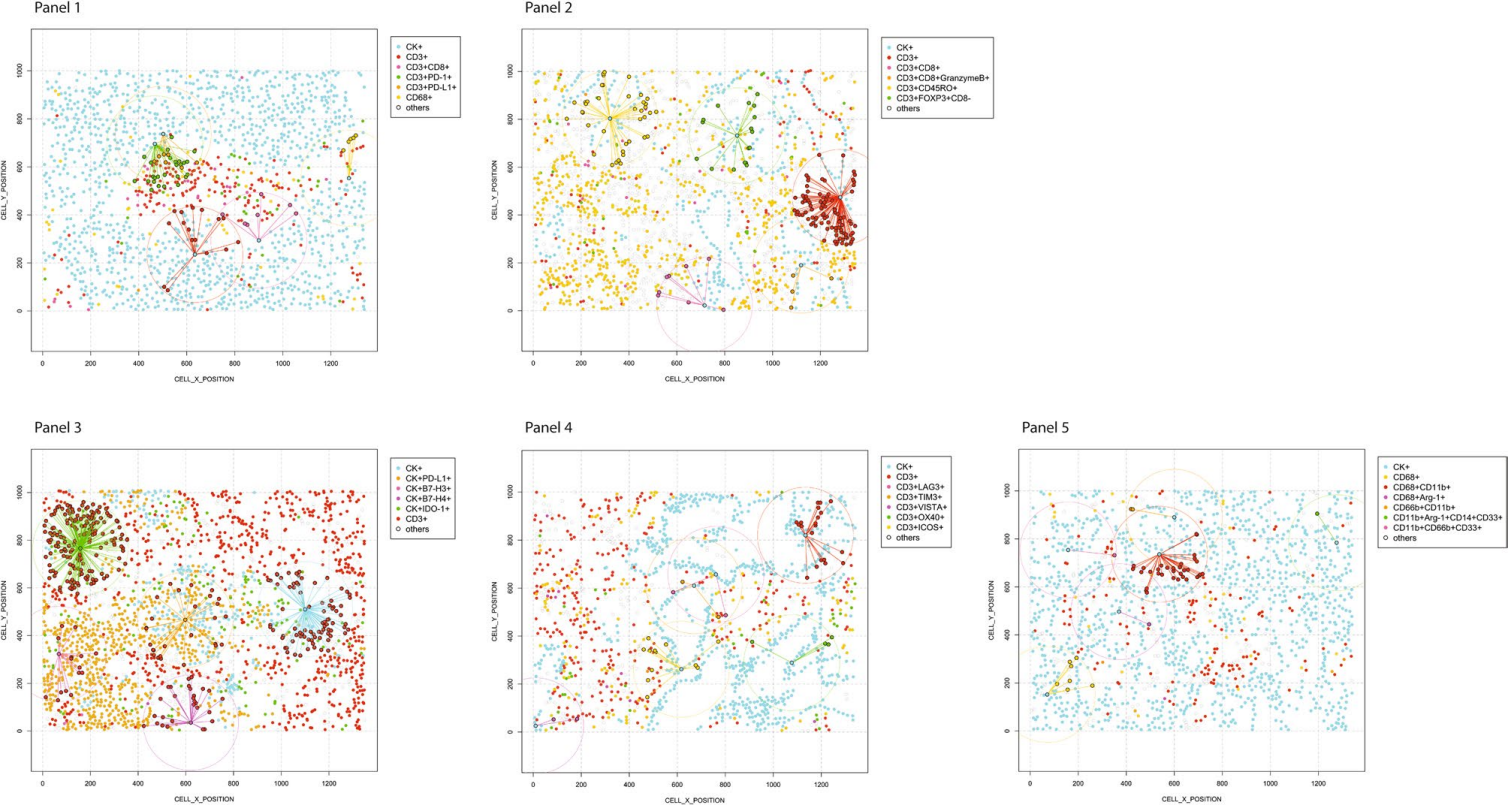
Spatial analysis showing (panels 1 and 2) representative examples of distance measurements from CK+ malignant cells (*cyan dots*) to different CD3+T-cell phenotypes (*identified by different colored dots according the marker’s expression*) and CD68+ macrophages (*yellow dots*); (panel 3) representative examples of distance measurements from CK+ malignant cells expressing different immune checkpoint markers (PD-L1, B7-H3, B7-H4, and IDO-1, *identified by different colored dots*) to CD3+T-cells (*red dots*); (panel 4) representative examples of distance measurements from CK+ malignant cells to CD3+T-cells expressing different immune checkpoint markers (LAG-3, TIM3, VISTA, OX40, and ICOS); and (panel 5) representative examples of distance measurements from CK+ malignant cells to different CD68+ macrophage phenotypes and other myeloid suppressor cell phenotypes using the X and Y coordinates with a radius of 200 microns (*circles*). The images were generated using R studio software version 3.6.1.

**Figure 6:**
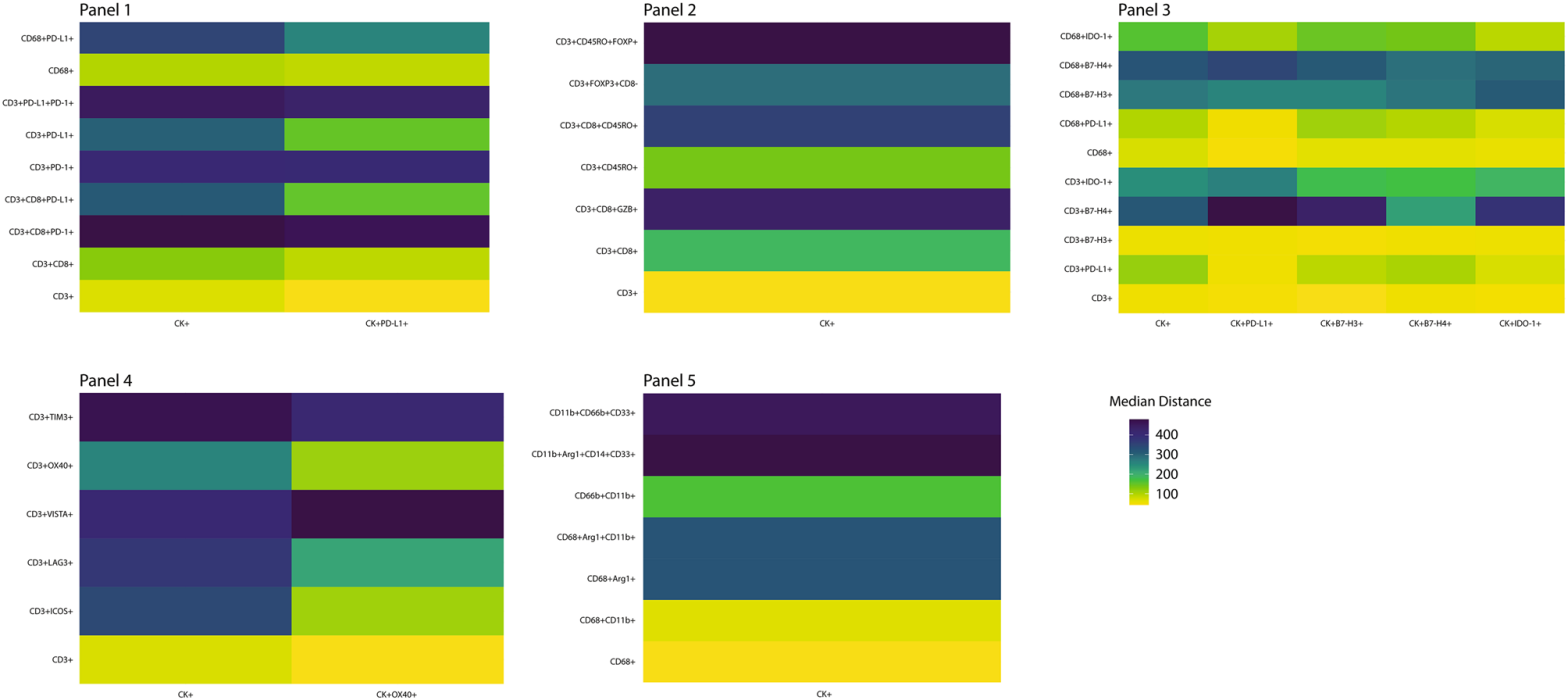
Heatmaps characterizing the median proximity from CK+ malignant cells and malignant cells expressing different immune checkpoint markers according the multiplex immunofluorescence panels. The images were generated using R studio software version 3.6.1.

The data from the image analysis of the mIF panels gave us the opportunity to explore the spatial distribution of the observed cell phenotypes according to their relationship with MCs (CK+). To map the spatial organization of the cells, we graphed their distribution using the X and Y coordinates of each cell phenotype in the NSCLC samples. The matrix of cell interaction was generated, and with that we were able to identify the median distance from MCs to the different cell phenotypes (Fig. 5). Using this matrix, we observed that the median distance between MCs and the others cell phenotypes across the panels was 241.96 microns. With that, we were able to consider all cells inside this distance radius as close to MCs, and the cells outside this radius as far from MCs. In that way, most of the CD3+T-cell phenotypes that play a role in the activation or regulation of the tumor microenvironment were observed as close to the MCs, while most of the CD3+T-cell immune checkpoints expressed were observed as far from the MCs in this cohort, (Fig. 6 and Supplementary Table 4). Interestingly, we observed that MCs that expressed PD-L1 were closer to cytotoxic T-cells (median distance, 78.86 microns) than were MCs that did not express PD-L1 (median distance, 125.82 microns). When we looked at the MCs expressing the other immune checkpoint markers, we found that CD3+T-cells were closer to CK+OX40+MCs (median distant, 38.29 microns) than to MCs expressing PD-L1 (median distant, 46.07 microns), B7-H3 (median distant, 39.02 microns), B7-H4 (median distant, 54.39 microns) or IDO-1 (median distant, 49.30 microns).

### Patterns of distribution

**Figure 7:**
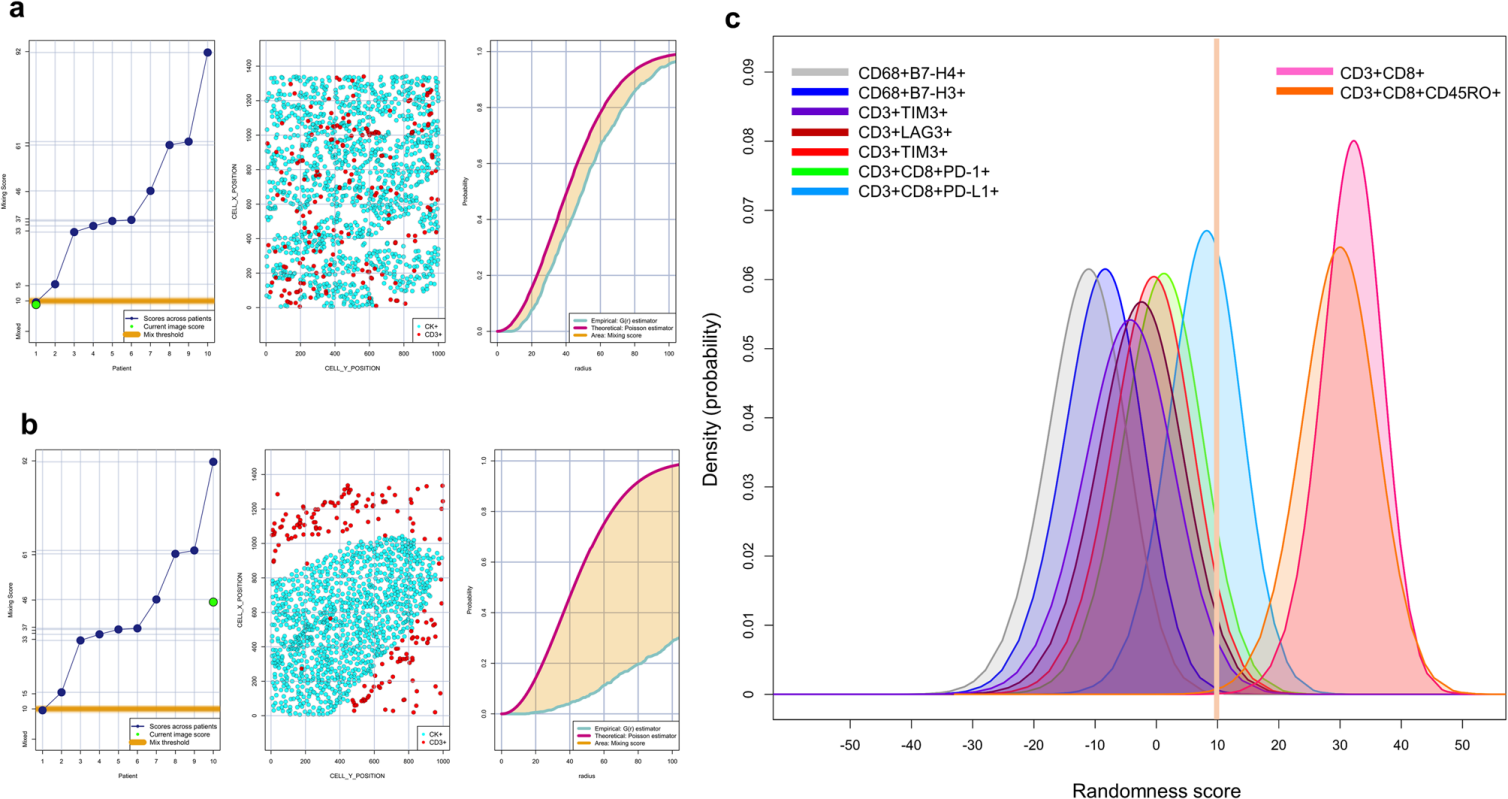
Individual nearest neighbor distance G function and theoretical Poisson curves graphics for the identification of different cellular patterns of distance distribution from CK+ malignant cells to CD3+T-cells and graphic representation of the predominate cell phenotypes that characterize those patterns. (a and b) The scoring scale across the cases and the threshold to be considered mixed or unmixed pattern (*left*), graphic distribution representation of the CD3+T-cells and CK+cells (*middle*), and the nearest neighbor distance G function and theoretical Poisson curves area (*right*). (**a**) Mixed pattern distribution of CD3+T-cells related to CK+cells, (**b**) unmixed pattern distribution of CD3+T-cells related to CK+cells, (c) graphical model showing the characterization of two different groups of interaction between specific phenotypes with malignant cells according their spatial distribution. Suppressor cell phenotypes have scores less than 10 and are characterized by a mixed pattern and more interaction with malignant cells, while cytotoxic T-cells (CD3+CD8+) and cytotoxic memory T-cells (CD3+CD8+CD45RO) have scores greater than 10 and are characterized by an unmixed pattern with less interaction to malignant cells. The images were generated using R studio software version 3.6.1.

Combining the empirically derived nearest neighbor distance G function curve from CD3+T-cells as a key marker to the theoretical Poisson function curve, we identified in our NSCLC cohort two patterns of distribution: mixed (closer to Poisson curve, from − 10 to + 10; Fig. 7a) and unmixed (farther from the Poisson curve, more than +10; Fig. 7b) independent of histology type. The mixed pattern was characterized by close interactions between CK+MCs and CD3+T-cells with a homogenous distribution between those cells, and it was observed in most of the samples. Interestingly, this pattern of interaction was related mostly with antigen-experienced cytotoxic T-cells expressing PD-1 and PD-L1, CD3+T-cells expressing LAG3 and TIM3, macrophages expressing B7-H3 and B7-H4, and polymorphonuclear myeloid-derived suppressor cells. In contrast, the unmixed pattern had low levels of interaction between CK+MCs and CD3+T-cells, showing MCs in cohesive nets with very little T-cell interaction, which was related predominantly to cytotoxic T-cells (CD3+CD8+) and cytotoxic memory T-cells (CD3+CD8+CD45RO+). These two patterns characterized the close interaction between MCs and predominant suppressor cells phenotypes in our samples (Fig. 7c). When we correlated these patterns with tumor recurrence in our samples, we observed that all cases with clinical information confirming recurrence showed the mixed pattern with predominant immunosuppressive cells populations; however, this finding did not reach statistical significance.

## Discussion

In this study, we optimized five automated mIF panels using TSA and a set of immune markers to identify immune checkpoints, myeloid-derived suppressor cells, and different tumor-associated immune cells that play an important role in the tumor microenvironment and can be relevant to translational pathology studies and new targeted therapies. We also applied these panels in a set of 10 NSCLC tissue samples to statistically assess the reproducibility of this technique, by quantifying and comparing different cell phenotypes between two time points, and to analyze the tumor microenvironment and the cellular spatial distribution of those different cell populations and their possible correlations with clinicopathologic characteristics. We observed a high analytic reproducibility of the cell phenotypes analyzed in each panel. The cell phenotypes showed high correlation both within samples (CV > 0.5) and across samples (CV > 0.7). The tumor microenvironment analysis showed a predominant T-cell suppressive activation via immune checkpoints expression predominantly via PD-L1/PD-1, and cases with tumor recurrence showed higher amounts of MCs expressing B7-H4 than did cases without recurrence. Another interesting finding was the different patterns of functional spatial distribution observed, with a predominant interaction between immunosuppressive cell populations and MCs shown by the mixed pattern.

In recent years, mIF has been shown to be an invaluable tool for tumor tissue immune profiling, enabling the identification of several targets in the same tissue section and the development of novel predictive biomarkers for cancer immunotherapy^9,21–23^. Characterization of the tumor microenvironment in cancer has become essential to following changes in immunologic phenotype inside the tumor, and this ability could enable the discovery of predictive biomarkers to be targeted with immunotherapies to guide the identification of new immunotherapeutic interventions^24–26^. These optimized mIF staining panels can be used to study the inflammatory tumor repertoire, immune checkpoints, and myeloid suppressor markers that have several implications for translational studies and thus have generated a new way to visualize and better understand the tumor microenvironment^24^.

Using a combination of single IF protocols, we can generate panels of six antibodies raised in the same species with simultaneous staining on a single paraffin tissue section^11,14^. The TSA system amplifies the signal of the primary antibody, which is especially useful in low-expression targets^27,28^; however, for the same reason it is not ideal for differentiating different levels of marker expression. The automated staining methodology can be performed in 1 day (14–17 h) for up to seven Opal fluorophore tyramides linked with their antibody plus DAPI in an autostainer, diminishing the time of staining (4–5 days) used in the published manual protocol^14^ and thus saving time and sources. Overall, we observed that these automated protocols can be handled easily, avoiding fluctuations in the level of specific signals from the marker used or from the background at the end of the optimization process, as is sometimes observed from human errors when the slides are stained manually. We also increased the area of the analyzed ROIs from the previously reported 0.334 mm^2^ (669 × 500 µm) with the Vectra scanner to 0.650 µm^2^ (931 × 698 µm) with 20 × resolution using the Vectra-Polaris 3.0.3 system. As was previously observed in the manual protocol^14^, the use of control tissues as well as diligent antibody optimization by IHC, single IF, and then mIF are essential steps for obtaining high-quality stains in manual and automated staining^19^. Additionally, we showed that proper balance of the different fluorophore tyramides linked with a specific antibody and maintenance of a specific range of exposure times, 50–150 ms, is fundamental to prevent cross-talking reactions or umbrella effects during the optimization process of any mIF panel^19,29^, especially during the construction of the spectral library.

Furthermore, using the automated staining, we were able to improve the previously published panels 1 and 2^14^ to make them immunologically more comprehensive by changing some antibodies, such as the not very stable CD4 for the PD-1 marker, thus completing the PD-L1/PD-1 axis in panel 1. In panel 2, we included CD3 and CD8 to obtain specific T-cell phenotypes such as activated cytotoxic T-cells (CD3+CD8+GranzymeB+), memory T-cells (CD3+CD45RO+), and regulatory T-cells (CD3+FOXP3+CD8-), all important cell phenotypes for activation and regulation of T-cells.

The analysis of tissue control sections by IHC and mIF revealed similar histologic patterns of marker expression and showed the importance of controls to compare individual antibodies by IHC and IF, as well as to identify the exact location and pattern of distribution of the different cell markers. As previously published^14,15^, PD-L1+ cells from control tonsil tissues show membranous expression of PD-L1 in epithelial crypts. Likewise, the other immune checkpoint markers—including B7-H3, B7-H4 (tested in the lung cancer tissue microarray positive control), VISTA, LAG3, ICOS, and TIM3—and immune checkpoint stimulatory markers, such as IDO-1 and OX40, showed similar patterns of cellular expression and distribution between IHC and mIF in tonsil and in lung cancer control tissues^18^. Similarly, the CK marker; T-cell markers such as CD3, CD8, granzyme B, PD-1, CD45RO, and FOXP3; and macrophage markers included in the myeloid panel, such as CD68, Arg-1, CD14, CD11b, CD33, and CD66b showed similar staining patterns and expression between IHC and mIF in tonsil positive control tissues. Improvement of the previously published panels 1 and 2, and the introduction of panels 3, 4, and 5, gave us the ability to study numerous specific cell phenotypes and to show the advantages of this technique as well as its potential for translational cancer research and longitudinal studies.

In our cohort of NSCLC samples, we were able to identify various cell phenotypes at consistent levels across the panels, with a high analytic reproducibility within panels between different time points using sequential tissue sections. Overall, the studied cell phenotypes showed high correlation, both within samples and across samples (week 1 vs. week 2), demonstrating that the multiplexing of these biomarkers in five panels following our protocol was successful. We believe that this finding also demonstrates the practical scalability of this method. As expected, cell phenotypes with low frequency or heterogeneous distribution, such as CK+PD-L1+, CD68+, CD3+IDO-1+, and CD3+VISTA+, showed lower correlation either within samples or across samples when normalized by the total number of cells from the corresponding ROIs, suggesting that the biological heterogeneous expression of these markers and the geographic distribution and number of selected ROIs contribute to differences in the densities of these cell phenotypes between panels. Therefore, the ROIs must be selected carefully and appropriately to represent all the different areas in the entire tumor section. This need can be addressed by the development of guidelines to standardize the selection of ROIs in this type of assay. The highest number of cell phenotypes observed across the panel—some of which were not included in the analysis—showed the biological cell heterogeneity in the microenvironment of those tumors and the possibility to discover new cells co-expressing those markers.

The analysis of the NSCLC cohort showed an immunosuppressive tumor microenvironment characterized principally by the axis of PD-L1/PD-1 and their interaction with different cellular subpopulations; this axis continues to be an important therapeutic target in many types of cancer^5^. Although memory T-cells and cytotoxic T-cells were observed in high densities compared with other cell populations, various cell phenotypes, such as regulatory T-cells, antigen-experienced T-cells expressing PD-L1, and T-cells expressing B7-H3, may have played a suppressive role, as suggested by their correlation with patient prognosis^30–32^. Importantly, we also showed the diversity of MCs simultaneously expressing different immune inhibitory checkpoints and stimulators as PD-L1, B7-H3, B7-H4, and IDO-1. this observation confirms our previous finding using single IHC^18^ and suggests that cancer can avoid immune surveillance using different pathways. The characterization of those markers and their recent association with prognosis opens the possibility of their becoming new therapeutic targets^33–37^. Most interestingly, the expression of B7-H4 by MCs was correlated with recurrence status in our cohort. As described previously, B7-H4 has been reported to be highly expressed in NSCLC^38^ and to promote malignant transformation, tumor growth, and metastasis^39^. Furthermore, we found that not only T-cells but also MCs can express OX40, although this expression occurs in a smaller proportion of MCs. This finding opens the possibility of discovering new cell phenotypes using those panels and this methodology. Finally, specific myeloid cell populations that are related with a poor clinical outcome^40,41^ and an immunosuppressive tumor microenvironment^42,43^ were observed, although in our cohort we found no correlation with clinicopathologic characteristics.

By applying spatial analysis in an exploratory manner, we showed the capability of this type of data to map the spatial relationships between different cell phenotypes. With that we were able to distinguish different cell populations that play specific roles in activation and regulation close to the MCs, suggesting that those cells can play specific roles according to their distribution^44^. Interestingly, MCs with PD-L1 expression showed closer proximity to cytotoxic T-cells than did MCs that did not express PD-L1. Additionally, we found that polymorphonuclear myeloid-derived suppressor cells had a median distance far from MCs but had close interaction with the MCs. This is interesting because the presence of tumor-infiltrating myeloid-derived suppressor cells has been related to a worse overall prognosis^45^. Interestingly, we found that T-cells were closer to MCs expressing OX40 than to MCs expressing other checkpoint markers. Although there are no data regarding OX40 expression in MCs, we believe that this phenotype may play an important role in the cellular composition of the tumor microenvironment. Using the distance from MCs to T-cells with the G function and the theoretical curve we identified two different patterns of distribution and interactions in our samples. The mixed pattern was characterized by the close proximity and interaction between MCs and T-cells, predominantly antigen-experienced cytotoxic T-cells expressing PD-L1 and PD-1 and other inhibitory regulators such as LAG3 and TIM3; macrophages expressing B7-H3 and B7-H4; and polymorphonuclear myeloid-derived suppressor cells, suggesting that suppressive cells actively interface with the MCs and may increase the risk of tumor recurrence in those patients. In contrast, the unmixed pattern was related mostly to cytotoxic T-cells, and cytotoxic memory T-cells. The characterization of these two patterns suggests that combined inhibition of different pathways in addition to PD-L1/PD-1 might be an effective therapeutic strategy in NSCLC.

In summary, we demonstrated that this method of mIF staining, targeting different antibodies in the same tissue section, is reproducible when all the steps are carefully followed and can be a powerful tool with high-quality data. As several authors, including us, have described previously^19,46,47^, many parameters have significant effects during the optimization process of mIF staining: choosing reliable antibodies, determining the appropriate sequence of the targets, reagent concentrations, incubation times, blocking steps, fluorescence intensity harmonization, the use of fresh tissue sections for staining, and regular, thin cuts between 3 and 4 µm. Although we showed intrasite reproducibility, intersite reproducibility using large cohorts will also be required as this methodology is employed in multi-institutional studies^46,47^ and prior to clinical use. The immune suppressive microenvironment observed in our cohort of NSCLC included not only the PD-L1/PD-1 axis but also other inhibitory pathways, such as the LAG3, VISTA, and TIM3 pathways, and myeloid-derived suppressor cells. This heterogeneity may partially explain why only some patients respond to mono inhibitory therapies^48^. Comprehensive immuno-profiling using mIF panels will hopefully improve our understanding of how different factors can determine disease progression or resistance or response to immunotherapies and can help to determine new treatment approaches.

## Supplementary Information

Supplementary Information 1.

Supplementary Information 2.

Supplementary Information 3.

Supplementary Information 4.

Supplementary Information 5.

Supplementary Information 6.

Supplementary Information 7.

Supplementary Information 8.

Supplementary Information 9.

Supplementary Information 10.

Supplementary Information 11.

Supplementary Information 12.

Supplementary Information 13.

Supplementary Information 14.

Supplementary Information 15.

Supplementary Information 16.

Supplementary Information 17.

Supplementary Information 18.

Supplementary Information 19.

Supplementary Information 20.

## Data Availability

The raw datasets used and/or analyzed during the current study are available from the corresponding author on reasonable request.

## Abbreviations

ADC: Adenocarcinoma
CK: Cytokeratin
CV: Coefficient of variation
DAPI: 4′,6-diamidino-2-phenylindole
IHC: Immunohistochemistry
MC: Malignant cell
mIF: Multiplex immunofluorescence
NSCLC: Non–small cell lung cancer
PD-1: Programmed cell death protein 1
ROI: Region of interest
SCC: Squamous cell carcinoma
