# Supplementary material for: Immuno-profiling and cellular spatial analysis using five immune oncology multiplex immunofluorescence panels for paraffin tumor tissue
Source: Sci Rep. 2021 Apr 19;11:8511. doi: 10.1038/s41598-021-88156-0 (PMC8055659; doi:10.1038/s41598-021-88156-0)
Supplement: Supplementary file 1 — Supplementary Information 1. [file 41598_2021_88156_MOESM1_ESM.docx]

**Immuno-Profiling and Cellular Spatial Analysis Using Five Immune Oncology Multiplex Immunofluorescence Panels for Paraffin Tumor Tissue**

Edwin Roger Parra, Maria C. Ferrufino-Schmidt, Auriole Tamegnon, Jiexin Zhang, Luisa Solis, Mei Jiang, Heladio Ibarguen, Cara Haymaker, J. Jack Lee, Chantale Bernatchez, Ignacio Ivan Wistuba.

**Legends of Supplementary Figures**

**Supplementary Figure 1.** Microphotographs of spectral library acquisition showing fluorescence extraction from the individual fluorophores used in the panels. The images were generated using Vectra/Polaris 3.0.3 scanner system and InForm 2.4.8 image analysis software (Akoya Biosciences).

**Supplementary Figure 2.** Microphotographs of representative examples of multiplex immunofluorescence scanning in low magnification (4×) and selected of regions of interest (ROI) by week and panel. Each rectangle represents an ROI selection from this case as an example. The images were generated using Vectra/Polaris 3.0.3 scanner system and visualized using the Phenochart 1.0.9 image viewer software (Akoya Biosciences),

**Supplementary Figure 3.** Microphotographs of optimization from individual immunohistochemistry (IHC) and multiplex immunofluorescence (mIF) of representative samples. Shown are the similar distribution patterns between individual markers by IHC (*top of each panel*) and each individual marker by the mIF (*bottom of each panel*) from tissue controls. A composite image of mIF is also shown on the *right* of each panel. 20× magnification. Panel 3 includes a representative example of lung cancer simultaneously expressing B7-H3 and B7-H4, which is not frequently observed in tonsil controls. The images were generated using Vectra/Polaris 3.0.3 scanner system and InForm 2.4.8 image analysis software (Akoya Biosciences).

**Supplementary Figure 4.** Boxplots obtained by dividing the cell phenotype studded by the total number of cells, showing various phenotypes from panel 1 at week 1 and week 2 and the corresponding scatter plots with linear regression lines for each sample. The images were generated using R studio software version 3.6.0.

**Supplementary Figure 5.** Boxplots obtained by dividing the cell phenotype studded by the total number of cells, showing various phenotypes from panel 2 at week 1 and week 2 and the corresponding scatter plots with linear regression lines for each sample. The images were generated using R studio software version 3.6.0.

**Supplementary Figure 6.** Boxplots obtained by dividing the cell phenotype studded by the total number of cells, showing various phenotypes from panel 3 at week 1 and week 2 and the corresponding scatter plots with linear regression lines for each sample. The images were generated using R studio software version 3.6.0.

**Supplementary Figure 7.** Boxplots obtained by dividing the cell phenotype studded by the total number of cells, showing various phenotypes from panel 4 at week 1 and week 2 and the corresponding scatter plots with linear regression lines for each sample. The images were generated using R studio software version 3.6.0.

**Supplementary Figure 8.** Boxplots obtained by dividing the cell phenotype studded by the total number of cells, showing various phenotypes from panel 5 at week 1 and week 2 and the corresponding scatter plots with linear regression lines for each sample. The images were generated using R studio software version 3.6.0.

**Supplementary Figure 9.** Trellis plots of phenotypes from panel 1 by ROI and week and obtained by dividing the cell phenotype studded by the total number of cells. The blue dots represent week 1 and the red dots represent week 2, visualizing consistency over time for each marker in panel 1 from all the samples. The images were generated using R studio software version 3.6.0.

**Supplementary Figure 10.** Trellis plots of phenotypes from panel 2 by ROI and week and obtained by dividing the cell phenotype studded by the total number of cells. The blue dots represent week 1 and the red dots represent week 2, visualizing consistency over time for each marker tested from all samples. The images were generated using R studio software version 3.6.0.

**Supplementary Figure 11.** Trellis plots of phenotypes from panel 3 by ROI and week and obtained by dividing the cell phenotype studded by the total number of cells. The blue dots represent week 1 and the red dots represent week 2, visualizing consistency over time for each marker tested from all samples. The images were generated using R studio software version 3.6.0.

**Supplementary Figure 12.** Trellis plots of phenotypes from panel 4 by ROI and week and obtained by dividing the cell phenotype studded by the total number of cells. The blue dots represent week 1 and the red dots represent week 2, visualizing consistency over time for each marker tested from all samples. The images were generated using R studio software version 3.6.0.

**Supplementary Figure 13.** Trellis plots of phenotypes from panel 5 by ROI and week and obtained by dividing the cell phenotype studded by the total number of cells. The blue dots represent week 1 and the red dots represent week 2, visualizing consistency over time for each marker tested from all samples. The images were generated using R studio software version 3.6.0.

**Supplementary Figure 14.** Boxplots showing the CK+ phenotypes for each week from the different panels and obtained by dividing the cell phenotype studded by the total number of cells. Median percentages of these two phenotypes differ between panels but not between weeks. The images were generated using R studio software version 3.6.0.

**Supplementary Figure 15.** Boxplots showing the CD3+ phenotypes for each week from the different panels and obtained by dividing the cell phenotype studded by the total number of cells. Median percentages of these two phenotypes differ between panels but not between weeks. The images were generated using R studio software version 3.6.0.
