# Supplementary figures and images for: Immuno-profiling and cellular spatial analysis using five immune oncology multiplex immunofluorescence panels for paraffin tumor tissue
Source: Sci Rep. 2021 Apr 19;11:8511. doi: 10.1038/s41598-021-88156-0 (PMC8055659; doi:10.1038/s41598-021-88156-0)

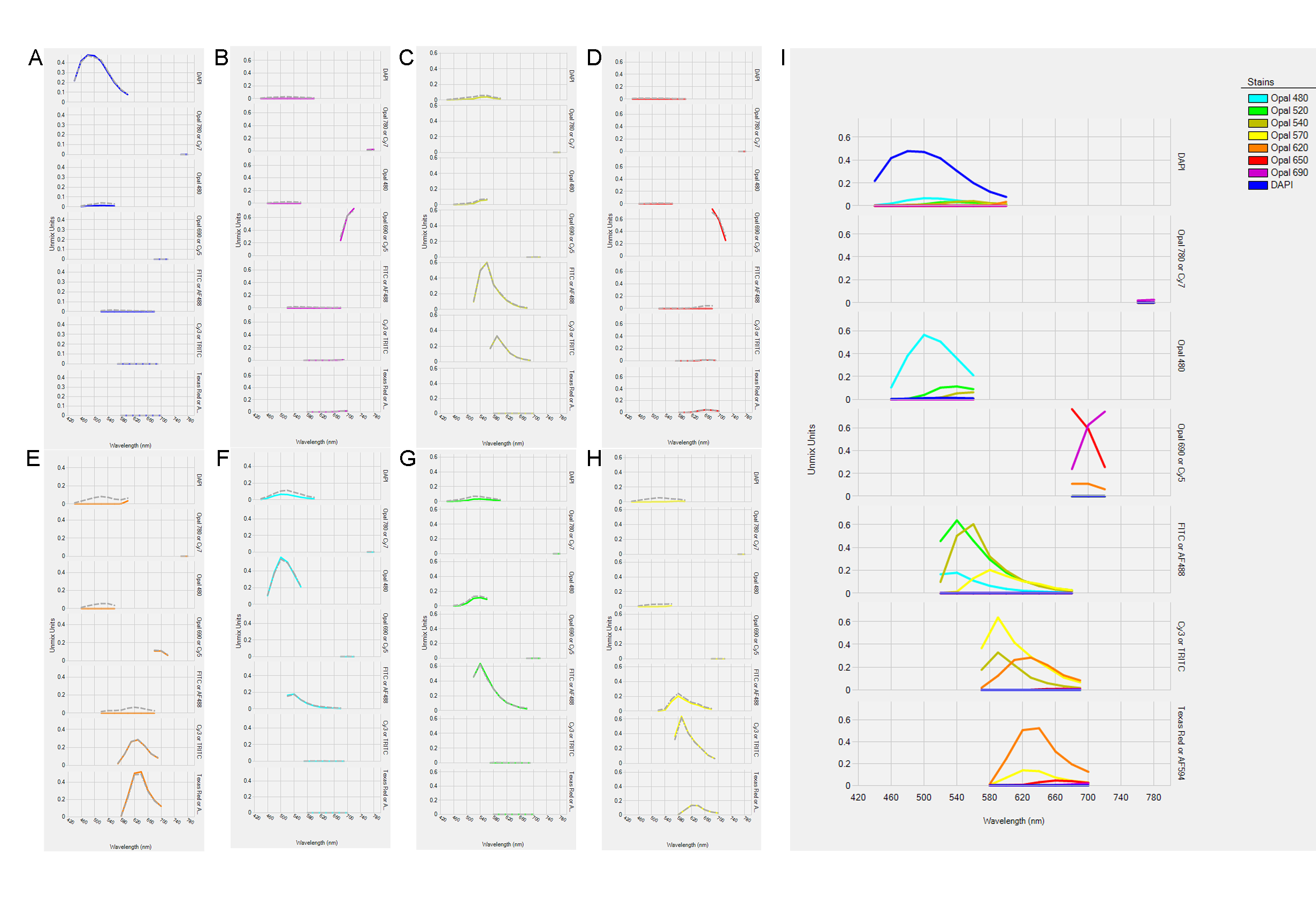

Supplement: Supplementary file 2 — Supplementary Information 2. [file 41598_2021_88156_MOESM2_ESM.tif]

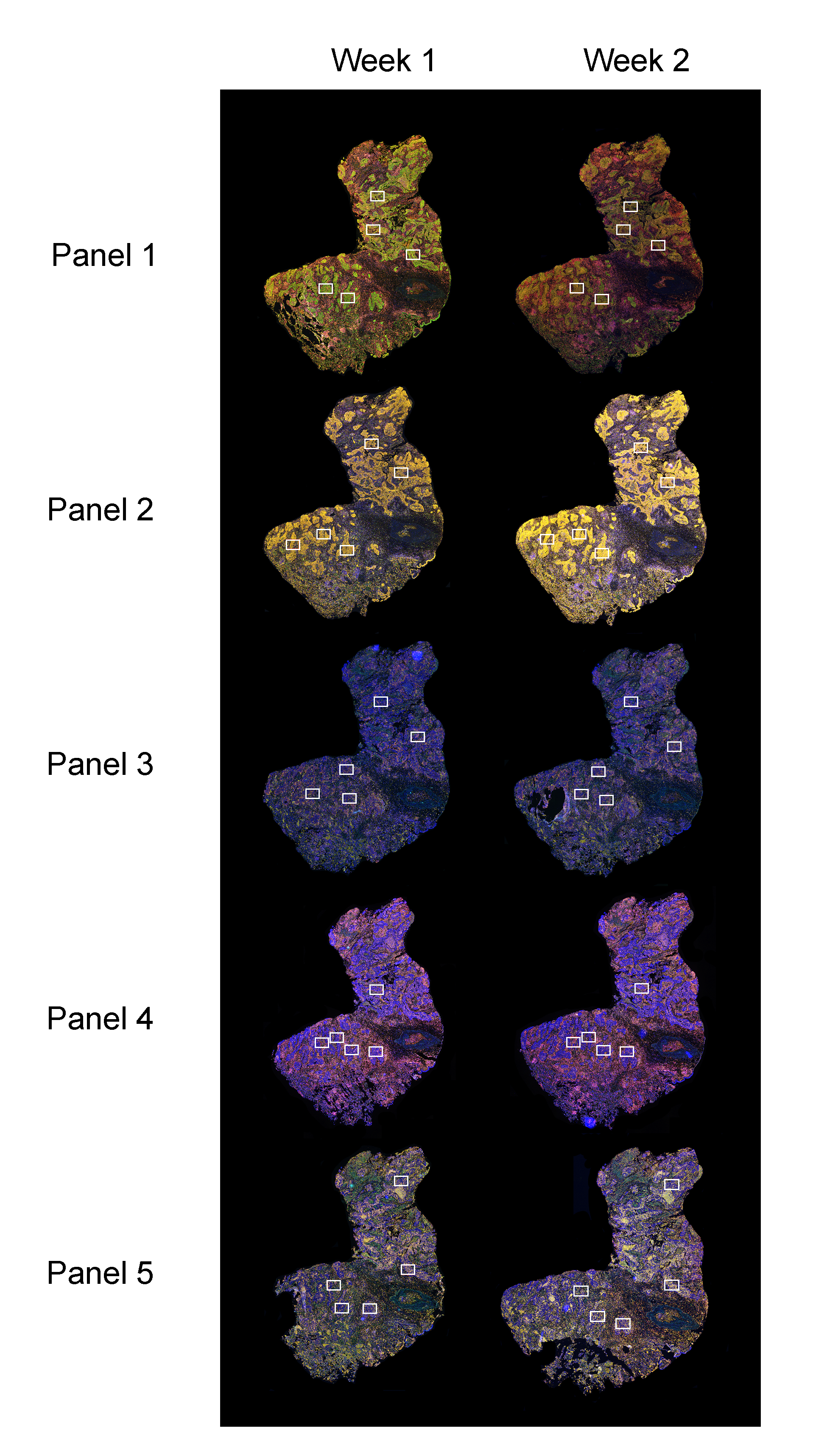

Supplement: Supplementary file 3 — Supplementary Information 3. [file 41598_2021_88156_MOESM3_ESM.tif]

Panel 1

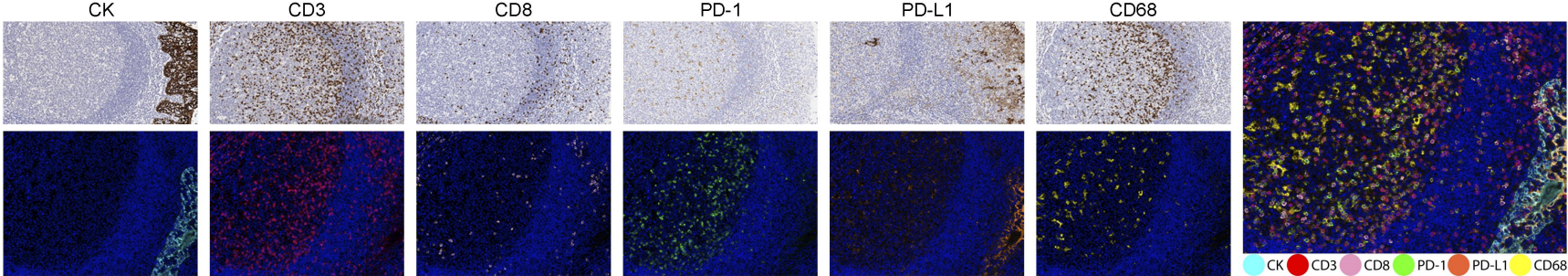

Panel 2

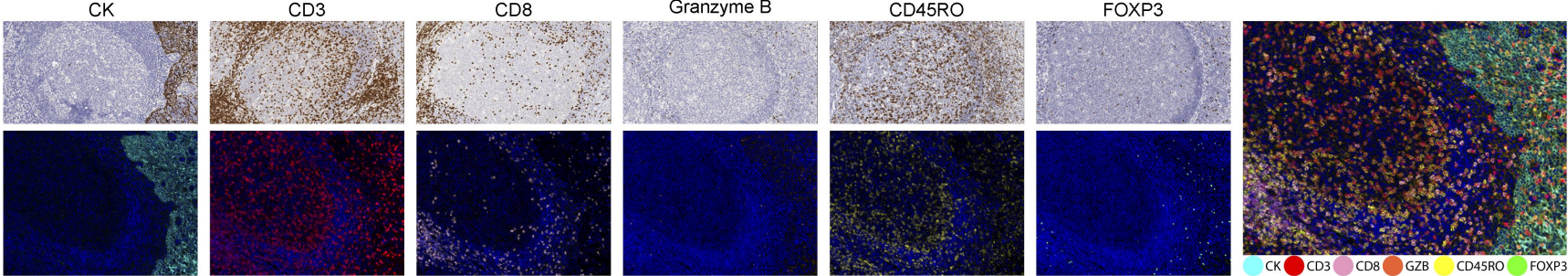

Panel 3

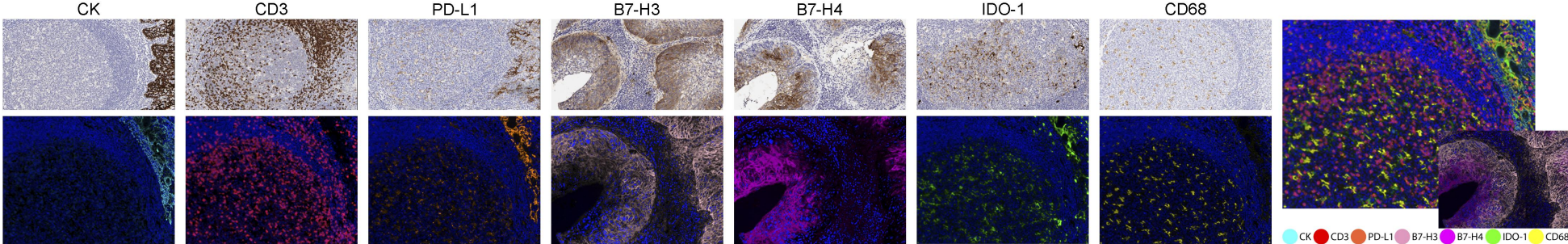

Panel 4

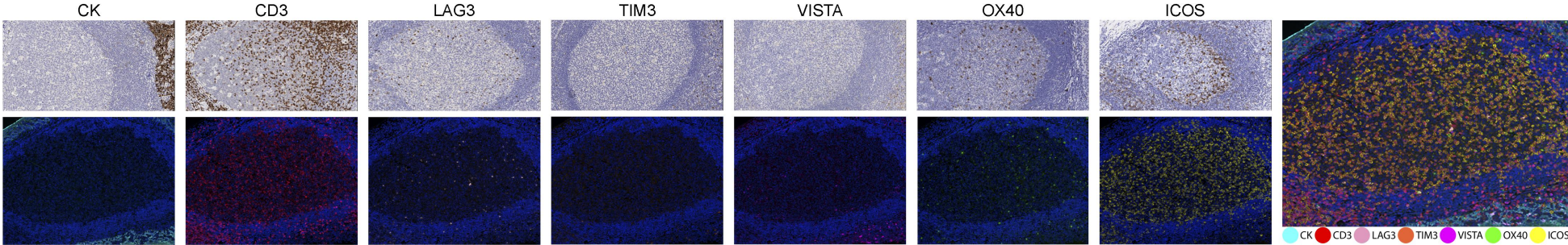

Panel 5

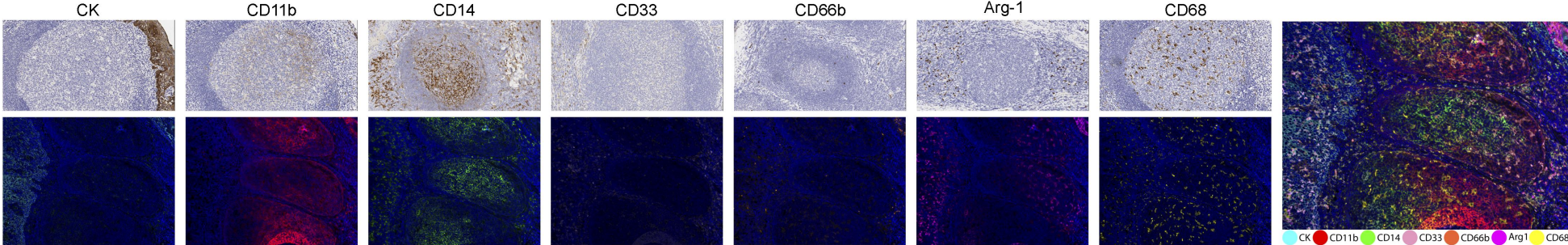

Supplement: Supplementary file 4 — Supplementary Information 4. [file 41598_2021_88156_MOESM4_ESM.pdf]

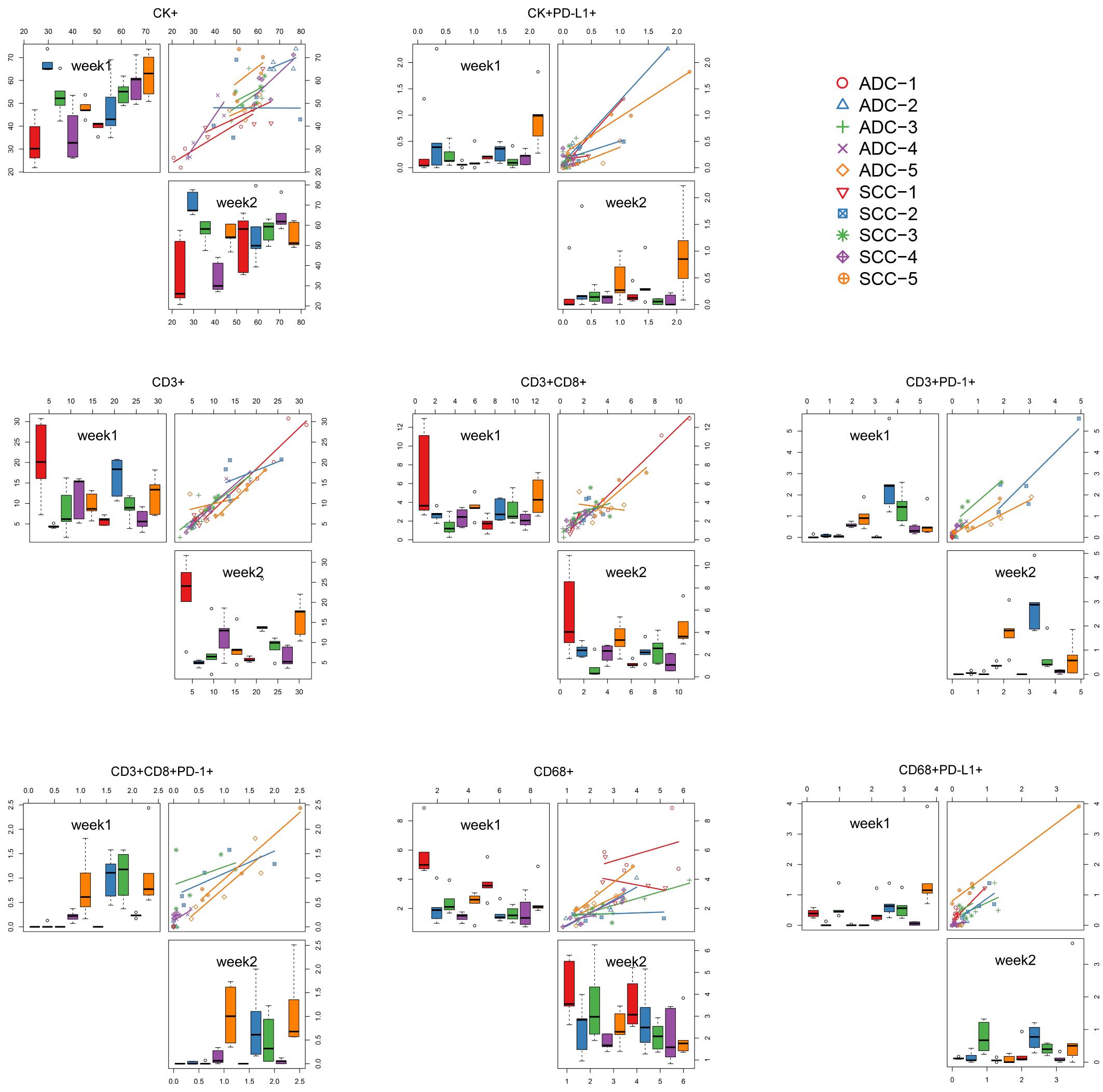

Supplement: Supplementary file 5 — Supplementary Information 5. [file 41598_2021_88156_MOESM5_ESM.tif]

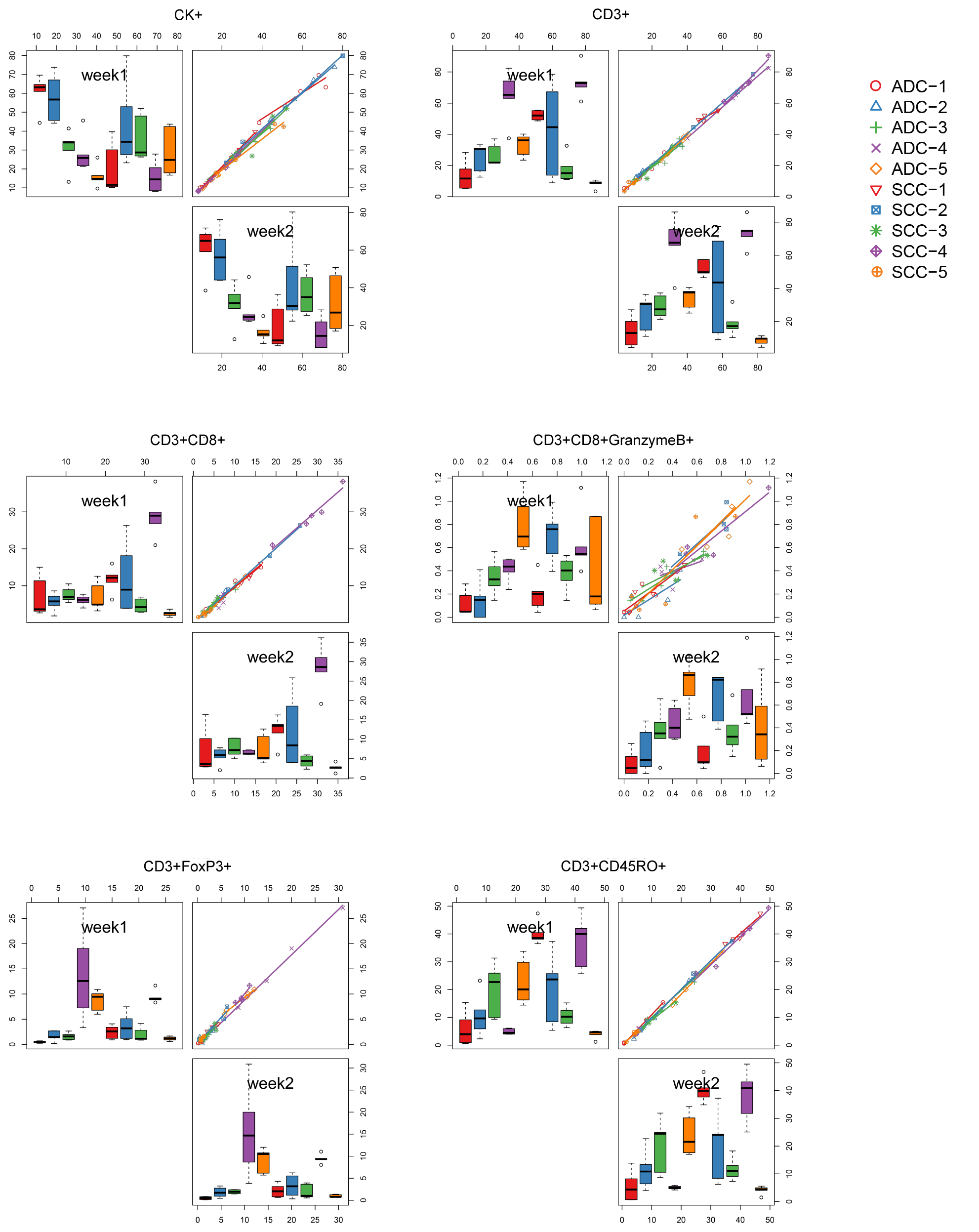

Supplement: Supplementary file 6 — Supplementary Information 6. [file 41598_2021_88156_MOESM6_ESM.tif]

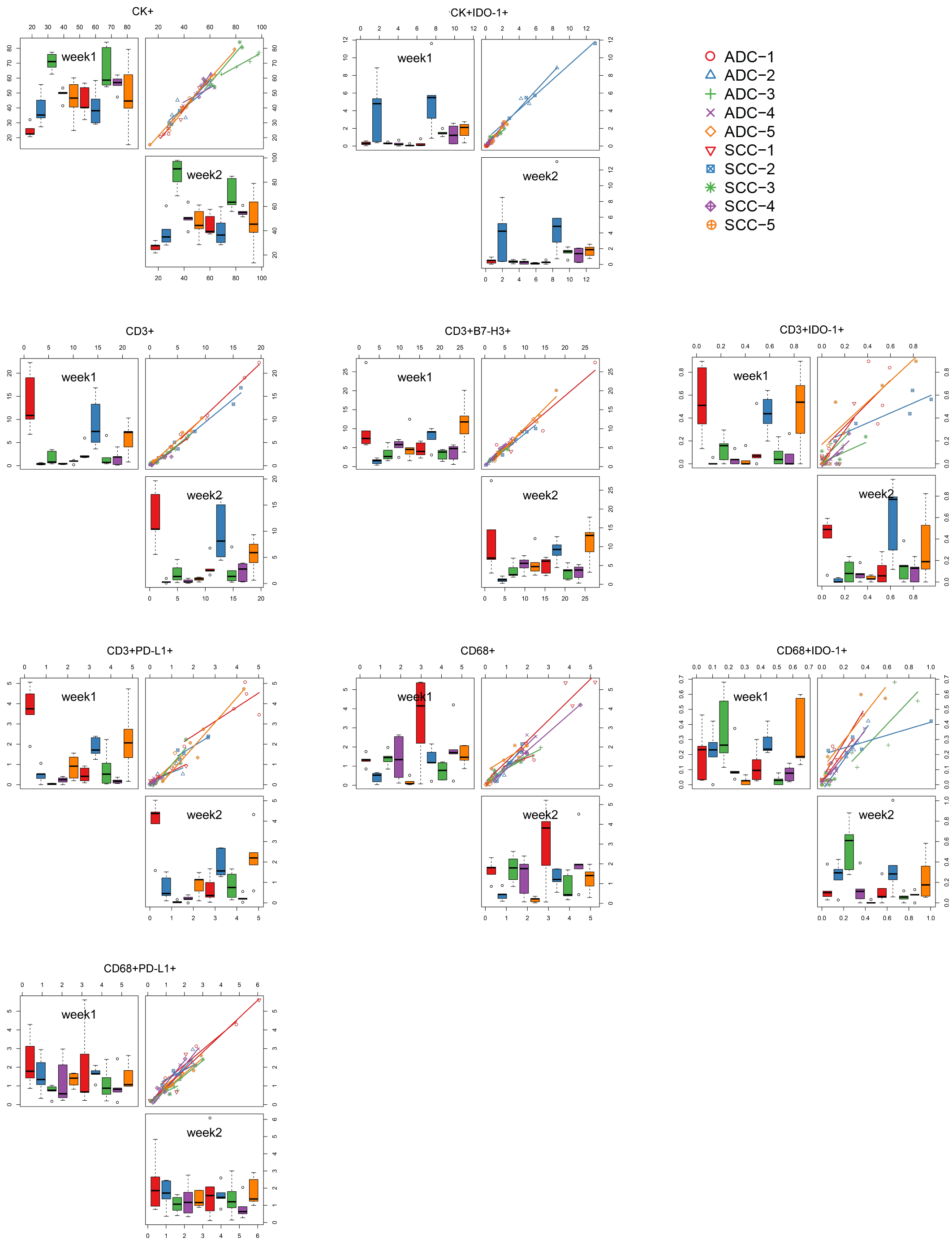

Supplement: Supplementary file 7 — Supplementary Information 7. [file 41598_2021_88156_MOESM7_ESM.tif]

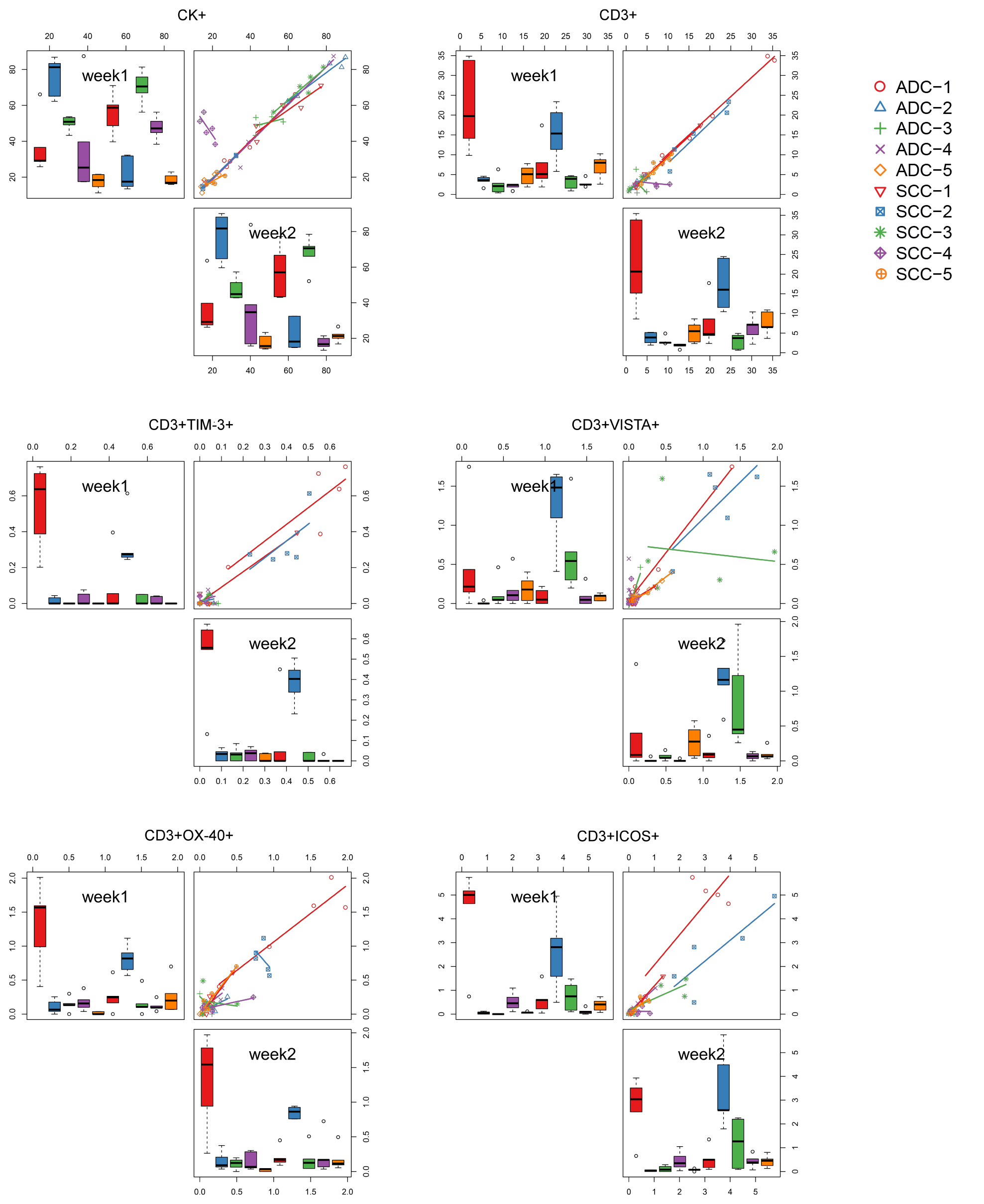

Supplement: Supplementary file 8 — Supplementary Information 8. [file 41598_2021_88156_MOESM8_ESM.tif]

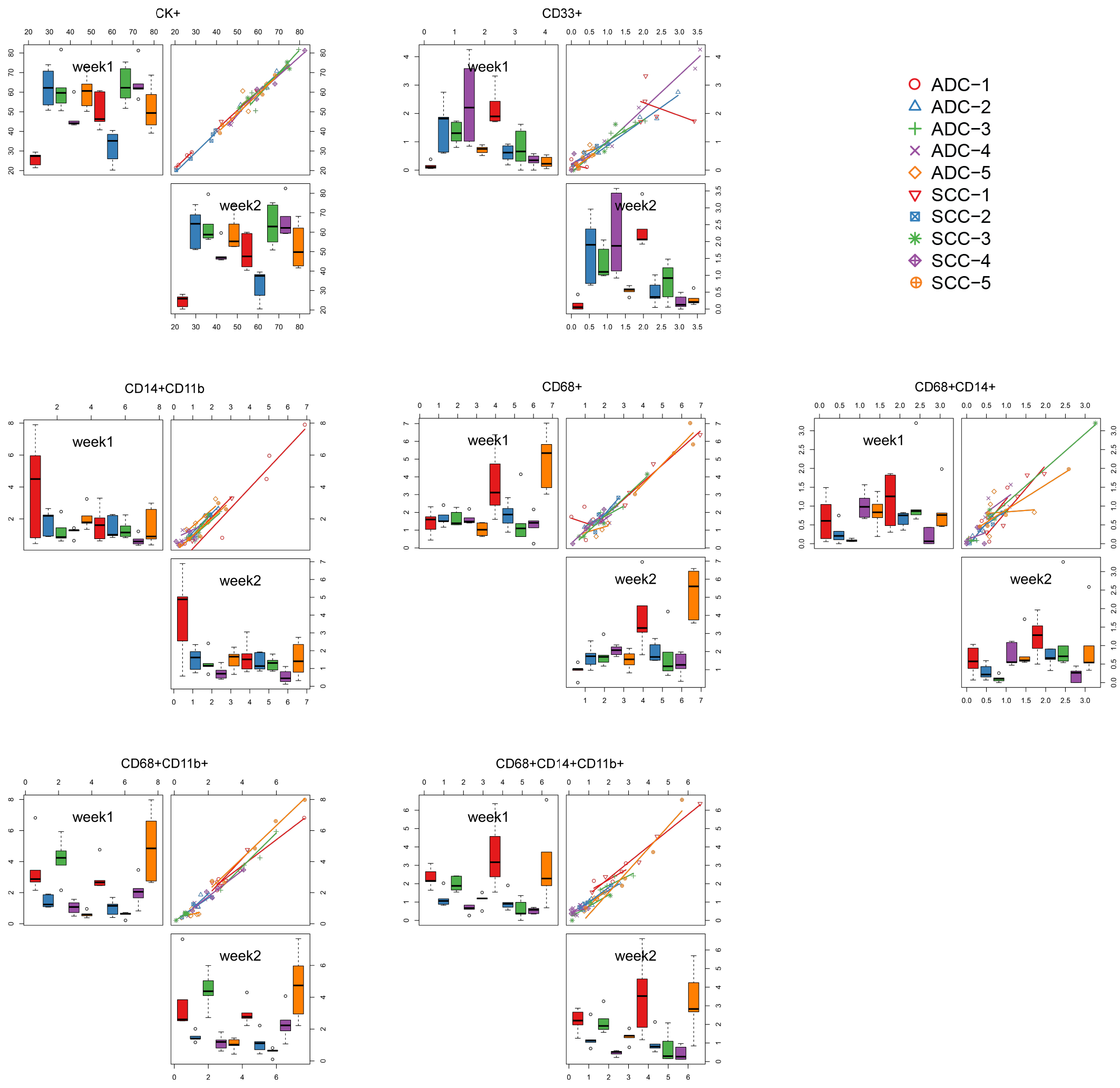

Supplement: Supplementary file 9 — Supplementary Information 9. [file 41598_2021_88156_MOESM9_ESM.tif]

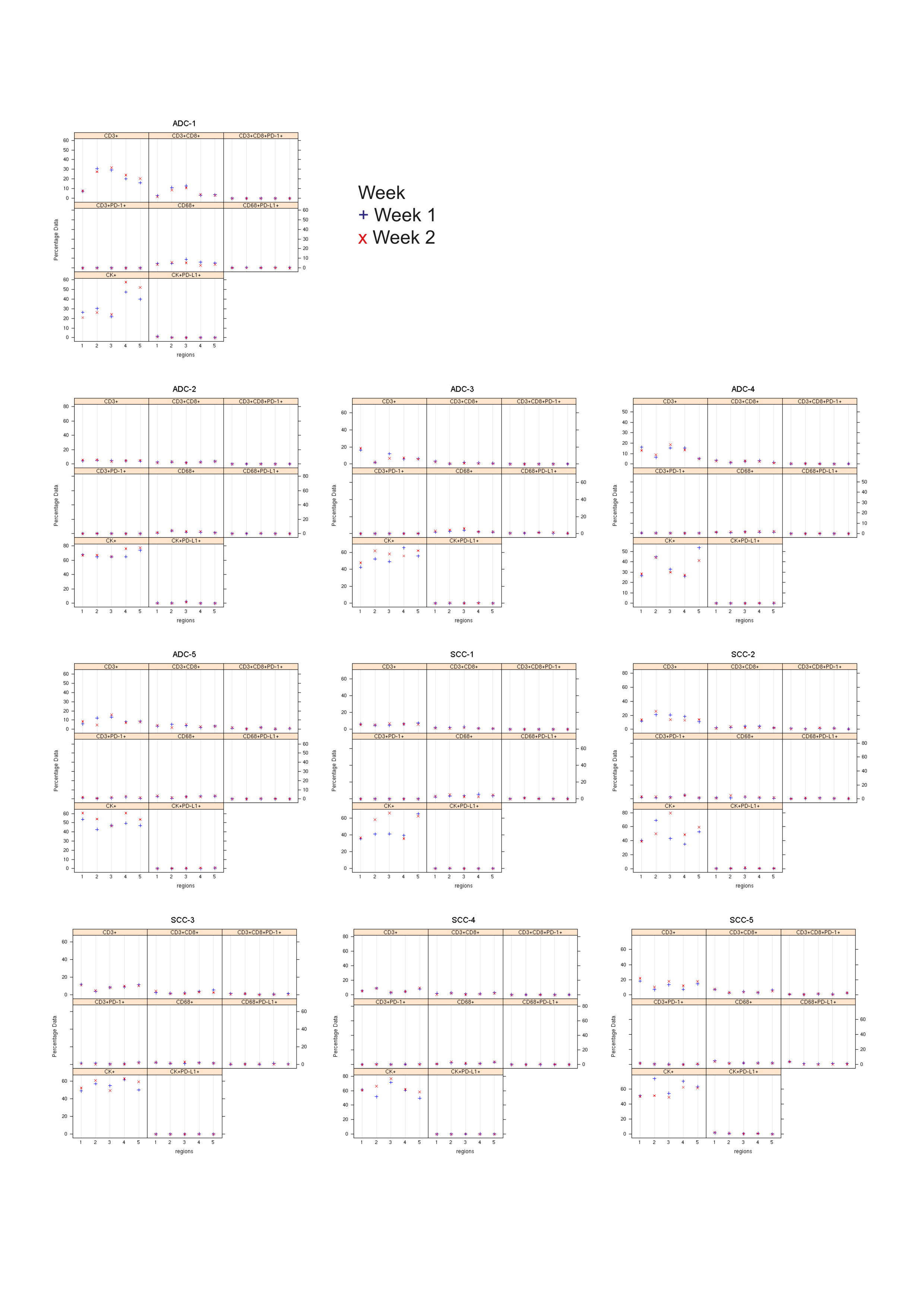

Supplement: Supplementary file 10 — Supplementary Information 10. [file 41598_2021_88156_MOESM10_ESM.tif]

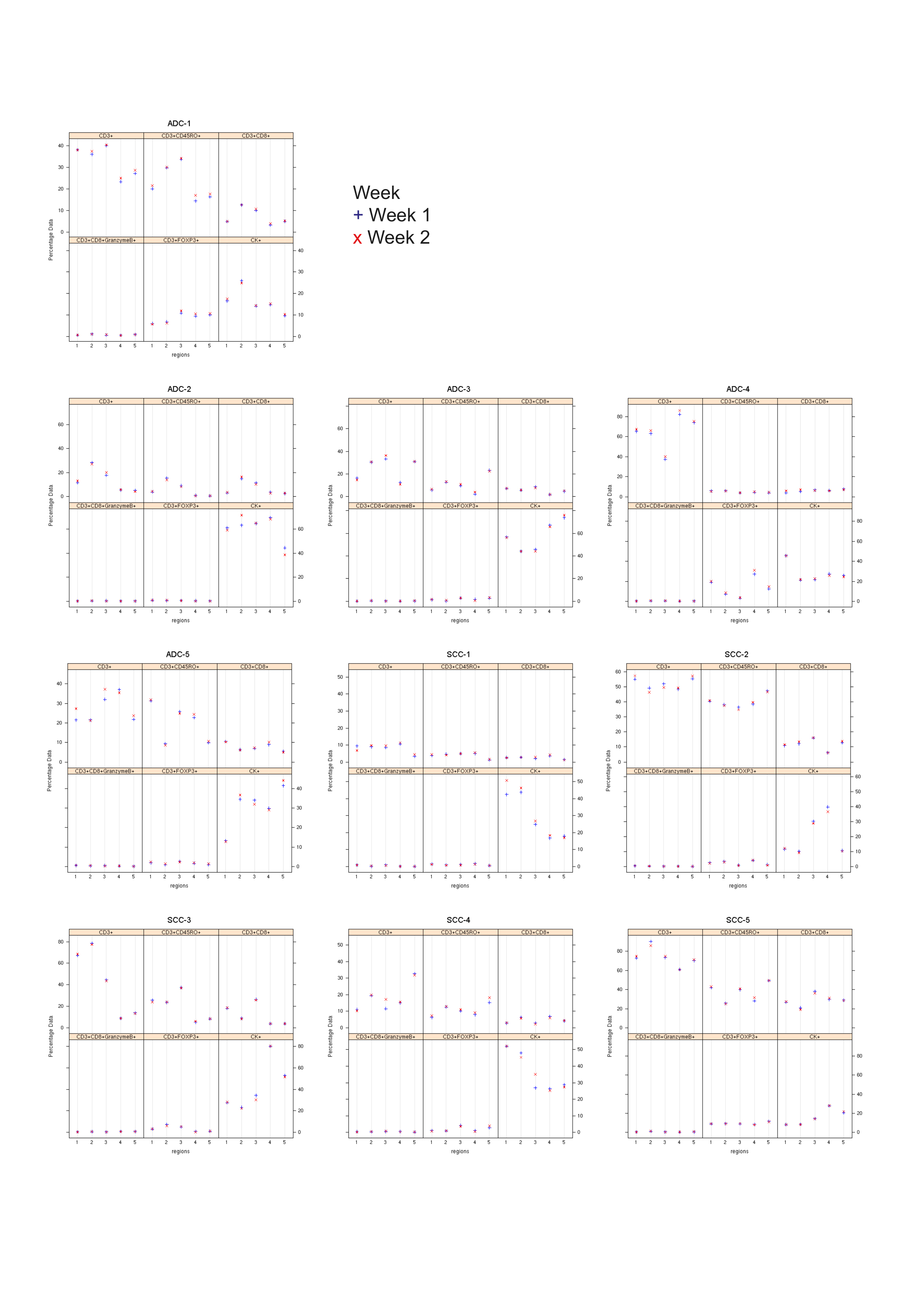

Supplement: Supplementary file 11 — Supplementary Information 11. [file 41598_2021_88156_MOESM11_ESM.tif]

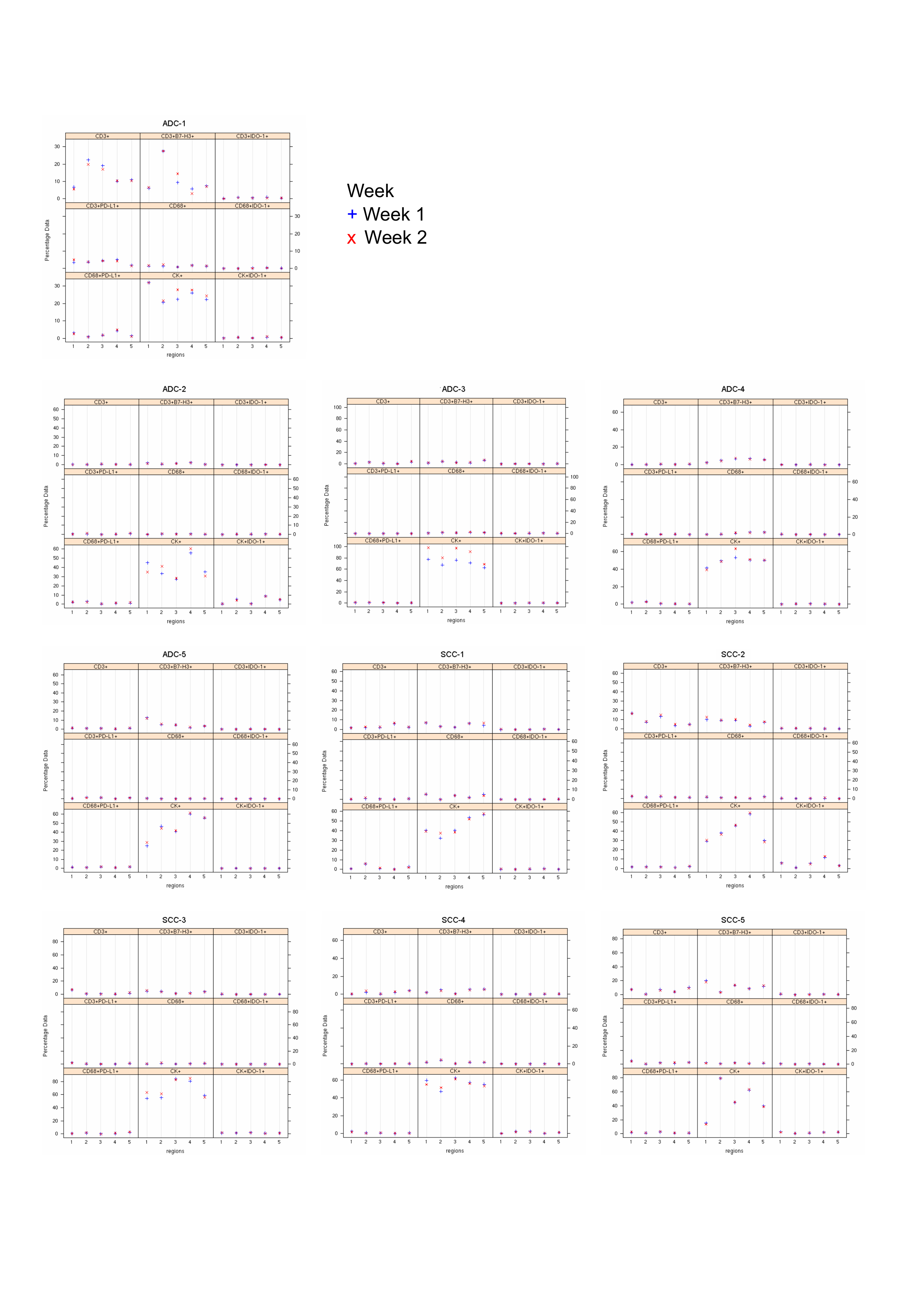

Supplement: Supplementary file 12 — Supplementary Information 12. [file 41598_2021_88156_MOESM12_ESM.tif]

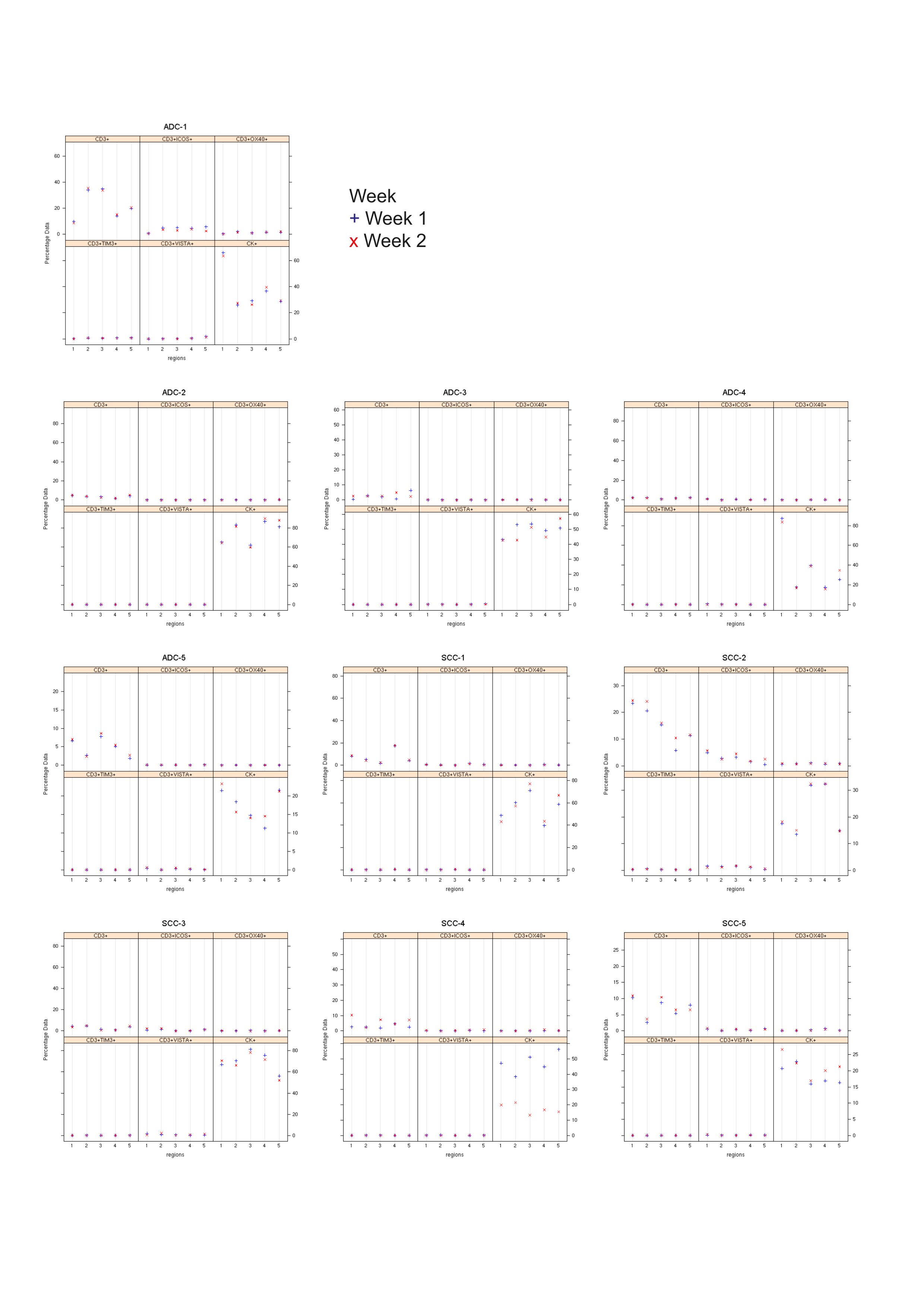

Supplement: Supplementary file 13 — Supplementary Information 13. [file 41598_2021_88156_MOESM13_ESM.tif]

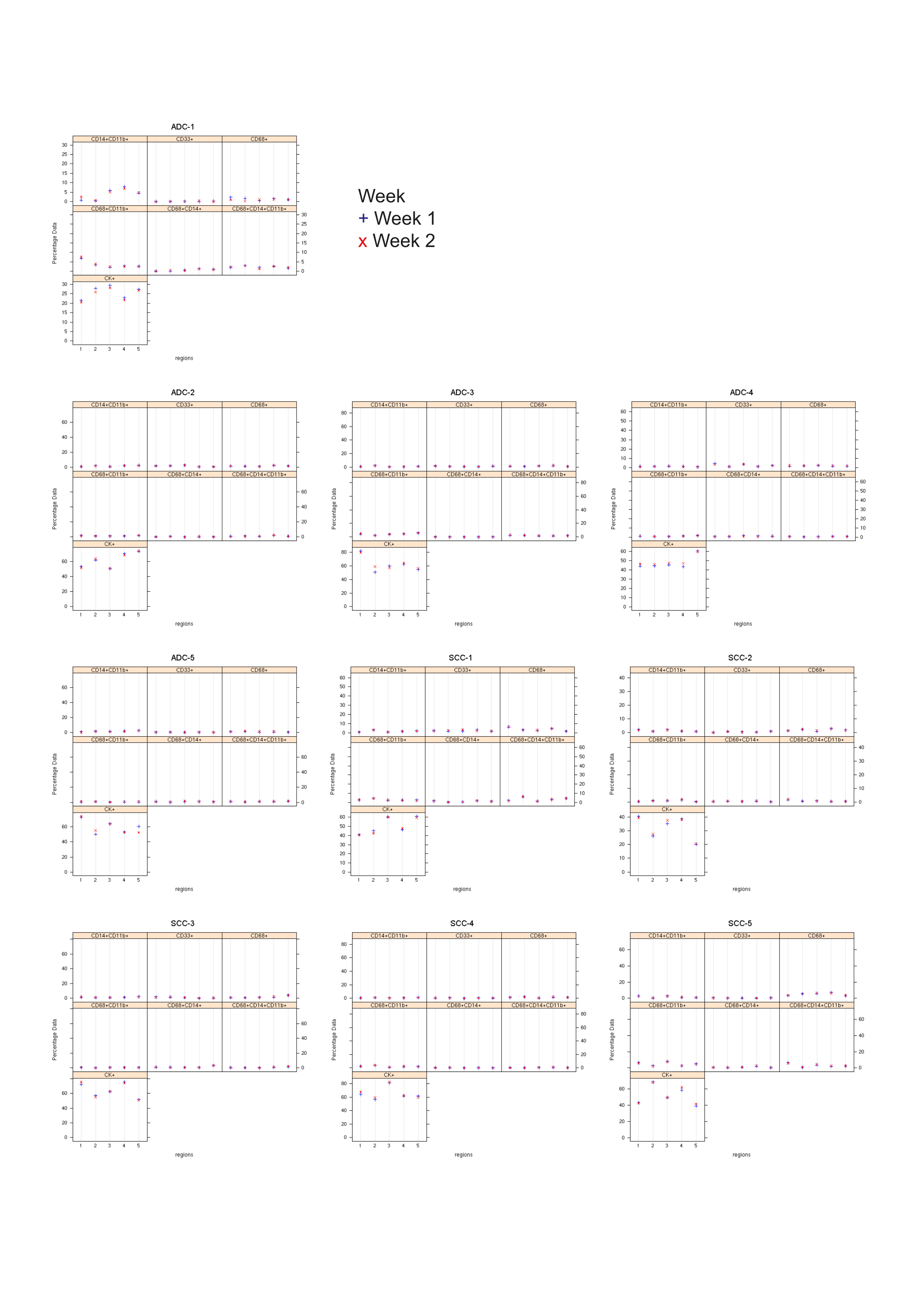

Supplement: Supplementary file 14 — Supplementary Information 14. [file 41598_2021_88156_MOESM14_ESM.tif]

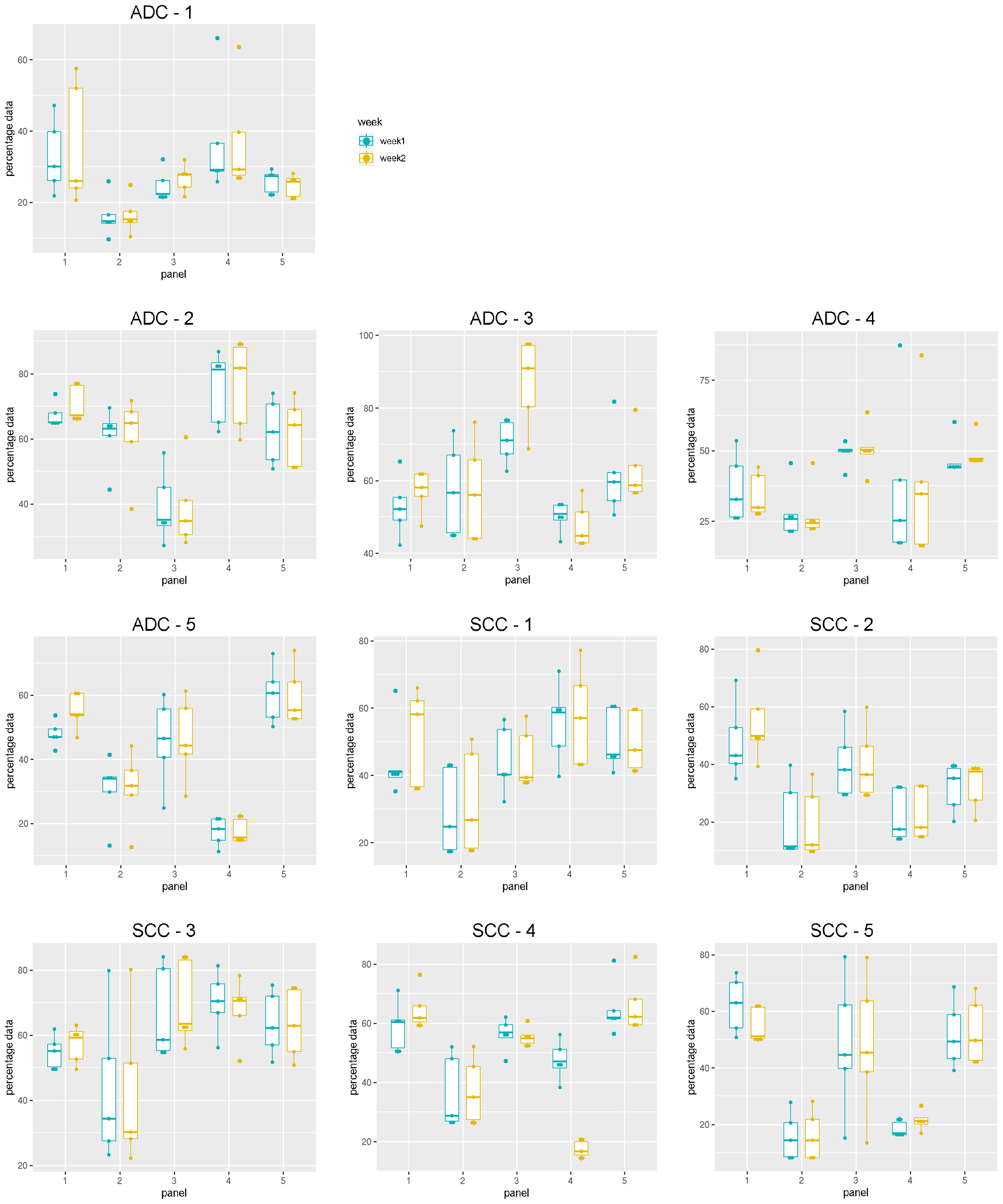

Supplement: Supplementary file 15 — Supplementary Information 15. [file 41598_2021_88156_MOESM15_ESM.tif]

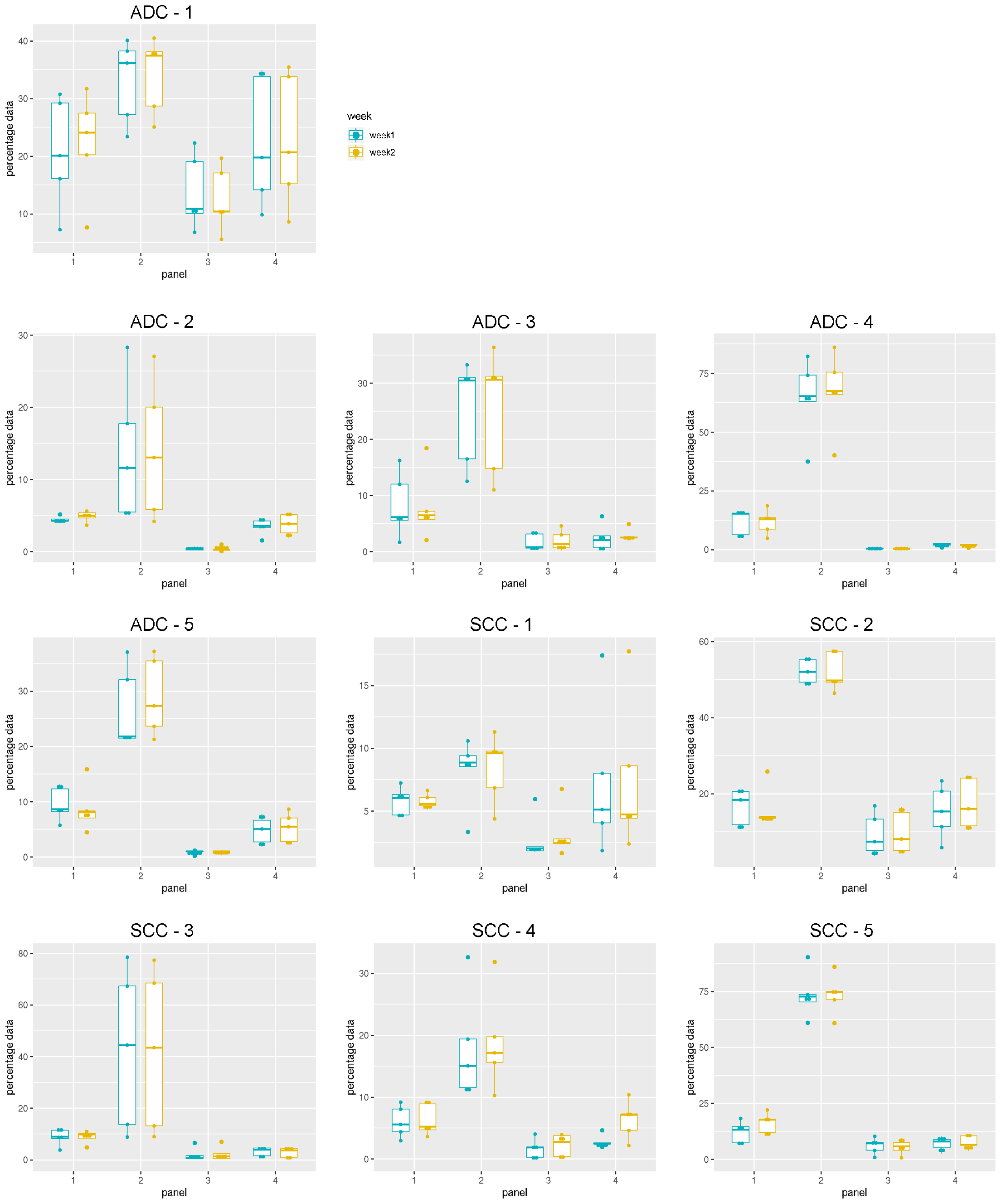

Supplement: Supplementary file 16 — Supplementary Information 16. [file 41598_2021_88156_MOESM16_ESM.tif]
