# Supplementary material for: Immuno-profiling and cellular spatial analysis using five immune oncology multiplex immunofluorescence panels for paraffin tumor tissue
Source: Sci Rep. 2021 Apr 19;11:8511. doi: 10.1038/s41598-021-88156-0 (PMC8055659; doi:10.1038/s41598-021-88156-0)
Supplement: Supplementary file 17 — Supplementary Information 17. [file 41598_2021_88156_MOESM17_ESM.docx]

**Immuno-Profiling and Cellular Spatial Analysis Using Five Immune Oncology Multiplex Immunofluorescence Panels for Paraffin Tumor Tissue**

Edwin Roger Parra, Maria C. Ferrufino-Schmidt, Auriole Tamegnon, Jiexin Zhang, Luisa Solis, Mei Jiang, Heladio Ibarguen, Cara Haymaker, J. Jack Lee, Chantale Bernatchez, Ignacio Ivan Wistuba.

**Supplementary Table 1.** Antibody optimization by immunohistochemistry.

| **Antibody** | **Clone** | **Vendor** | **AR** | **Dilution** |
| --- | --- | --- | --- | --- |
| CD3 | A045201-2* | DAKO | PH6 | 1:100 |
| CD8 | C8/144B | Thermo Scientific | PH6 | 1:25 |
| CD45RO | UCHL1 | Leica Biosystems (RTU) | PH6 | RTU |
| GZB | 11F1 | Leica Biosystems (RTU) | PH9 | RTU |
| FOXP3 | D2W8E | Cell Signaling | PH9 | 1:100 |
| PD-1 | [EPR4877(2)] | ABCAM | PH6 | 1:250 |
| PD-L1 | E1L3N | Cell Signaling | PH6 | 1:100 |
| B7-H3 | D9M2L | Cell Signaling | PH9 | 1:80 |
| B7-H4 | D1M8I | Cell Signaling | PH9 | 1:100 |
| IDO-1 | SP260 | Spring | PH6 | 1:400 |
| ICOS | D1K2T | Cell Signaling | PH6 | 1:100 |
| LAG3 | D2G4O | Cell Signaling | PH6 | 1:100 |
| OX40 | ACT-3 | Affimetrix (eBioscience) | PH6 | 1:50 |
| TIM3 | D5D5R | Cell Signaling | PH9 | 1:100 |
| VISTA | D1L2G | Cell Signaling | PH9 | 1:200 |
| Arg-1 | D4E3M | Cell Signaling | PH6 | 1:400 |
| CD11b | EPR1344 | ABCAM | PH6 | 1:8000 |
| CD14 | SP192 | ABCAM | PH9 | 1:400 |
| CD33 | [PWS44 (M)] | Leica Biosystems | PH9 | 1:100 |
| CD66b | G10F5 | BioLegend | PH6 | 1:100 |
| CD68 | PG-M1 | DAKO | PH6 | 1:450 |
| CK | AE1/AE3 | DAKO | PH6 | 1:50 |

*Catalog number.

AR, antigen retrieval; GZB, granzyme B; CK, cytokeratin; RTU, ready to use.
