# Supplementary material for: Immuno-profiling and cellular spatial analysis using five immune oncology multiplex immunofluorescence panels for paraffin tumor tissue
Source: Sci Rep. 2021 Apr 19;11:8511. doi: 10.1038/s41598-021-88156-0 (PMC8055659; doi:10.1038/s41598-021-88156-0)
Supplement: Supplementary file 18 — Supplementary Information 18. [file 41598_2021_88156_MOESM18_ESM.docx]

**Supplementary Table 2.** Clinicopathologic characteristics of patients with non-small cell lung cancer (N=10).

| **Variable** | **Category** | **Number (%)** |
| --- | --- | --- |
| Age at diagnosis, y | Median (Range) | 66 (54-76) |
| Gender | Male | 3 (30%) |
|  | Female | 7 (70%) |
| Smoking history | No | 1 (10%) |
|  | Yes | 9 (90%) |
| Tumor status (AJCC8)* | pT1 | 1 (10%) |
|  | pT2 | 5 (50%) |
|  | pT3 | 4 (40%) |
| Nodal status (AJCC8)* | pN0 | 3 (30%) |
|  | pN2 | 4 (40%) |
|  | pN3 | 4 (40%) |
| Stage (AJCC8)* | 1B | 3 (30%) |
|  | 2 | 3 (30%) |
|  | 3B | 4 (40%) |
| Tumor size | ADC (median) | 3.0 cm |
|  | SCC (median) | 2.7 cm |
| Adjuvant therapy | No | 7 (70%) |
|  | Yes | 3 (50.0%) |
| Recurrence | No | 7 (70%) |
|  | Yes | 3 (30%) |
| Vital status | Dead | 1 (10%) |
|  | Alive | 9 (90%) |

*The 8th edition of American Joint Committee on Cancer (AJCC) staging system.

ADC, Adenocarcinoma; SCC, Squamous cell carcinoma.
