# Supplementary material for: Immuno-profiling and cellular spatial analysis using five immune oncology multiplex immunofluorescence panels for paraffin tumor tissue
Source: Sci Rep. 2021 Apr 19;11:8511. doi: 10.1038/s41598-021-88156-0 (PMC8055659; doi:10.1038/s41598-021-88156-0)
Supplement: Supplementary file 19 — Supplementary Information 19. [file 41598_2021_88156_MOESM19_ESM.docx]

**Supplementary Table 3.** Antibody optimization by multiplex immunofluorescence using the Opal fluorophores (Akoya Biosciences).

| **Panel** | **Antibody (Ab)** | **Clone** | **Vendor** | **AR** | **Ab. Dilution** | **F** | **F. Dilution** |
| --- | --- | --- | --- | --- | --- | --- | --- |
| **1** | CK | AE1/AE3 | DAKO | PH6 | 1:50 | 620 | 1:100 |
|  | CD3 | A045201-2* | DAKO | PH6 | 1:100 | 690 | 1:100 |
|  | CD8 | C8/144B | Thermo Scientific | PH6 | 1:25 | 540 | 1:100 |
|  | PD-1 | [EPR4877(2)] | ABCAM | PH9 | 1:3000 | 650 | 1:200 |
|  | PD-L1 | E1L3N | Cell Signaling | PH6 | 1:1000 | 570 | 1:100 |
|  | CD68 | PG-M1 | DAKO | PH6 | 1:25 | 520 | 1:100 |
| **2** | CK | AE1/AE3 | DAKO | PH6 | 1:50 | 620 | 1:100 |
|  | CD3 | A045201-2* | DAKO | PH9 | 1:100 | 690 | 1:100 |
|  | CD8 | C8/144B | Thermo Scientific | PH6 | 1:25 | 540 | 1:100 |
|  | CD45RO | UCHL1 | Leica Biosystems | PH6 | RTU | 520 | 1:100 |
|  | GZB | 11F1 | Leica Biosystems | PH9 | RTU | 570 | 1:100 |
|  | FOXP3 | D2W8E | Cell Signaling | PH6 | 1:50 | 650 | 1:200 |
| **3** | CK | AE1/AE3 | DAKO | PH6 | 1:25 | 690 | 1:100 |
|  | CD3 | A045201-2* | DAKO | PH6 | 1:200 | 650 | 1:300 |
|  | PD-L1 | E1L3N | Cell Signaling | PH6 | 1:3000 | 570 | 1:100 |
|  | B7-H3 | D9M2L | Cell Signaling | PH9 | 1:200 | 540 | 1:100 |
|  | B7-H4 | D1M8I | Cell Signaling | PH9 | 1:500 | 480 | 1:100 |
|  | IDO-1 | SP260 | ABCAM | PH6 | 1:800 | 620 | 1:100 |
|  | CD68 | PG-M1 | DAKO | PH6 | 1:50 | 520 | 1:100 |
| **4** | CK | AE1/AE3 | DAKO | PH9 | 1:25 | 480 | 1:100 |
|  | CD3 | A045201-2* | DAKO | PH6 | 1:200 | 690 | 1:100 |
|  | ICOS | D1K2T | Cell Signaling | PH6 | 1:200 | 520 | 1:100 |
|  | LAG3 | D2G4O | Cell Signaling | PH6 | 1:200 | 650 | 1:200 |
|  | OX40 | ACT-3 | Affimetrix (eBioscience) | PH6 | 1:10 | 570 | 1:100 |
|  | TIM3 | D5D5R | Cell Signaling | PH6 | 1:100 | 540 | 1:100 |
|  | VISTA | D1L2G | Cell Signaling | PH9 | 1:400 | 620 | 1:100 |
| **5** | CK | AE1/AE3 | DAKO | PH6 | 1:50 | 690 | 1:150 |
|  | Arg1 | D4E3M | Cell Signaling | PH6 | 1:250 | 620 | 1:100 |
|  | CD11b | EPR1344 | ABCAM | PH6 | 1:6000 | 650 | 1:100 |
|  | CD14 | SP192 | ABCAM | PH6 | 1:300 | 480 | 1:100 |
|  | CD33 | [PWS44 (M)] | Leica Biosystems | PH9 | 1:50 | 520 | 1:100 |
|  | CD66b | G10F5 | BioLegend | PH6 | 1:100 | 540 | 1:150 |
|  | CD68 | PG-M1 | DAKO | PH6 | 1:50 | 570 | 1:100 |

AR, antigen retrieval; GZB, granzyme B; CK, cytokeratin; and F, fluorophore.

*Catalog number.
