# Supplementary material for: Immuno-profiling and cellular spatial analysis using five immune oncology multiplex immunofluorescence panels for paraffin tumor tissue
Source: Sci Rep. 2021 Apr 19;11:8511. doi: 10.1038/s41598-021-88156-0 (PMC8055659; doi:10.1038/s41598-021-88156-0)
Supplement: Supplementary file 20 — Supplementary Information 20. [file 41598_2021_88156_MOESM20_ESM.docx]

**Supplementary Table 4.** Median distance from malignant cells (CK+) to the different cell phenotypes observed across the panels.

| **Cell phenotype** | **Distance from CK+ cells, microns*** |
| --- | --- |
| **Panel 1** |  |
| CD3+ | 69.03 |
| CD3+PD-L1+ | 303.60 |
| CD3+PD-1+ | 404.19 |
| CD3+PD-L1+PD-1+ | 450.71 |
| CD3+CD8+ | 125.82 |
| CD3+CD8+PD-L1+ | 312.81 |
| CD3+CD8+PD-1+ | 479.03 |
| CD68+ | 98.24 |
| CD68+PD-L1+ | 348.24 |
| **Panel 2** |  |
| CD3+ | 53.62 |
| CD3+FOXP3+CD8- | 176.24 |
| CD3+CD8+ | 126.77 |
| CD3+CD8+Granzyme B+ | 245.65 |
| CD3+CD45RO+ | 101.95 |
| CD3+CD45RO+FOXP3+ | 262.38 |
| CD3+CD8+CD45RO+ | 192.79 |
| **Panel 3** |  |
| CD3+ | 56.46 |
| CD3+PD-L1+ | 167.63 |
| CD3+B7-H3+ | 61.30 |
| CD3+B7-H4+ | 503.61 |
| CD3+IDO-1+ | 380.77 |
| CD68+ | 89.98 |
| CD68+PD-L1+ | 134.42 |
| CD68+B7-H3+ | 435.69 |
| CD68+B7-H4+ | 515.11 |
| CD68+IDO-1+ | 236.45 |
| **Panel 4** |  |
| CD3+ | 79.03 |
| CD3+ICOS+ | 355.00 |
| CD3+LAG3+ | 389.17 |
| CD3+VISTA+ | 419.52 |
| CD3+OX40+ | 268.28 |
| CD3+TIM3+ | 482.75 |
| **Panel 5** |  |
| CD68+ | 88.31 |
| CD68+CD11b+ | 120.74 |
| CD68+Arg1+ | 445.07 |
| CD66b+CD11b+ | 241.97 |
| CD11b+Arg1+CD14+CD33+ | 600.89 |
| CD11b+CD66b+CD33+ | 529.08 |

*Cells within 241.96 microns of malignant cells were considered close; cells outside this radius were considered far from malignant cells.
